# Specific SLC25 carriers regulate mitochondrial protein synthesis

**DOI:** 10.1126/sciadv.aeb0049

**Published:** 2026-02-25

**Authors:** Danielle L. Rudler, Laetitia A. Hughes, Martin S. King, Jessica Baker, Richard G. Lee, Andrianto P. Gandadireja, Anisha Sunil, Samuel V. Fagan, Blake Payne, Nicola Gray, Tim McCubbin, Edmund R. S. Kunji, Oliver Rackham, Aleksandra Filipovska

**Affiliations:** ^1^ The Kids Research Institute Australia, Northern Entrance, Perth Children’s Hospital, 15 Hospital Avenue, Nedlands, Western Australia 6009, Australia.; ^2^ ARC Centre of Excellence in Synthetic Biology, University of Western Australia, Crawley, Western Australia 6008, Australia.; ^3^ Medical Research Council Mitochondrial Biology Unit, University of Cambridge, Keith Peters Building, Cambridge Biomedical Campus, Hills Road, Cambridge CB2 0XY, UK.; ^4^ Curtin Medical School and Curtin Medical Research Institute, Curtin University, Bentley, Western Australia 6102, Australia.; ^5^ Australian National Phenome Centre, Centre for Computational and Systems Medicine, Health Futures Institute, Murdoch University, Perth, Western Australia, Australia.; ^6^ Australian Institute for Bioengineering and Nanotechnology, The University of Queensland, Brisbane, Queensland 4072, Australia.; ^7^ ARC Centre of Excellence in Synthetic Biology, The University of Queensland, Brisbane, Queensland 4072, Australia.

## Abstract

A genome-wide knockout screen identified members of the SLC25 family of mitochondrial carrier proteins as important regulators of the rate of de novo mitochondrial protein synthesis. To elucidate this relationship, we generated human cell knockouts for SLC25A25, SLC25A44, SLC25A45, and SLC25A48, which have been shown to exchange adenosine triphosphate-magnesium (ATP-Mg) and phosphate, branched-chain amino acids, methylated basic amino acids, and choline, respectively. Multiomic and functional analyses identified that these four carriers are crucial for mitochondrial translation, biogenesis and function of the oxidative phosphorylation system, as well as mitochondrial morphology. Thermostability screens showed that SLC25A48 is specifically stabilized by choline, and changes in the mitochondrial metabolome and lipidome indicated defects in choline biosynthetic pathways and remodeling of mitochondrial membranes, both consistent with SLC25A48 being a choline transporter. These results highlight the essential roles of specific SLC25 transporters in maintaining mitochondrial structure and function and show that impaired transport of branched-chain amino acids, methylated basic amino acids, ATP-Mg, and choline affects mitochondrial translation.

## Teaser

Mitochondrial carrier proteins control protein synthesis, dynamics, and energy function through selective metabolite transport.

## INTRODUCTION

Eukaryotic cells are divided into discrete metabolic environments by membrane-bound organelles. Many of these membranes are impermeable to solutes, and therefore the exchange of metabolites and ions is facilitated by transporters and channels that can be divided into four superfamilies: the adenosine triphosphate (ATP)–binding cassette (ABC) transporters, ATPases, ion channels, and solute carrier (SLC) proteins (*1*, *2*). There are 53 members of the mitochondrial solute carrier family 25 (SLC25) in humans, all of which are nuclear encoded and reside in the mitochondrial inner membrane, with the exception of three that are localized to the mitochondrial outer membrane and one in peroxisomes (*3*–*6*). The SLC25 family is involved in the transport of a diverse range of molecules including amino acids, nucleotides, vitamins, inorganic ions, carboxylic acids, and fatty acids. Most characterized SLC25 transporters act as antiporters by the counter-exchange of chemically related substrates, but there are also members that function as uniporters or proton/substrate symporters (*7*). Inhibitors that lock specific states have been used to show that these proteins have two conformational states: the cytoplasmic-open state (c-state) and matrix-open state (m-state) in which the substrate-binding site is accessible from the intermembrane space and the mitochondrial matrix, respectively (*7*). The substrate-binding mechanism has been experimentally determined for the mitochondrial ADP/ATP carrier (*8*). The shuttling of metabolites that occurs between the mitochondrial intermembrane space and matrix mediated by members of the SLC25 family is important for mitochondrial function, maintenance, and homeostasis, including oxidative phosphorylation (OXPHOS). Mutations in mitochondrial transporters have been shown to impair the movement of metabolites and consequently cell metabolism, causing diverse clinical presentations (*2*, *9*–*11*). Advances have been made toward characterizing the SLC25 carrier proteins, but the substrates of some have still not been identified. Moreover, the molecular effects of the characterized transporters on mitochondrial gene expression have not been explored systematically.

In addition to their role in ATP synthesis via OXPHOS, mitochondria are hubs of biosynthetic activity for fatty acid synthesis and oxidation, mitochondrial protein and amino acid synthesis, cell death regulation, reactive oxygen species (ROS) signaling, and detoxification via the urea cycle (*12*, *13*). Mitochondria contain their own genome, and the expression of the mitochondrially encoded proteins is essential for OXPHOS and cell metabolism as impaired translation has been shown to lead to diverse pathologies [reviewed in (*14*)]. However, the impact of mitochondrial metabolism on the protein expression machinery is less well understood. During amino acid starvation, mitochondrial turnover and amino acid catabolism are elevated to reprogram metabolic function (*15*, *16*), suggesting that the metabolic environment may influence mitochondrial protein synthesis. The fidelity of mitochondrial translation can either exacerbate or improve the response to metabolic stress in mice in a tissue-specific manner (*17*). It is clear that mitochondrial protein expression is required for the robustness of mitochondrial metabolism; however, the impact of metabolic flux between the mitochondria and the cytoplasm on translation is not clear.

Recently, we developed a genetic screen to identify cell regulators of mitochondrial protein synthesis (*18*) and here we note an enrichment of a significant number of SLC25 carriers, indicating the importance of metabolite exchange for mitochondrial translation. To investigate this relationship between solute transport and mitochondrial protein synthesis, we identified specific SLC25 proteins that led to mitochondrial translation defects and generated human cell line knockouts of four members of the SLC25 family. We show that mitochondrial carriers SLC25A25, SLC25A44, SLC25A45, and SLC25A48 are required for mitochondrial translation and mitochondrial morphology, as their loss perturbs energy metabolism. We identified that SLC25A48 binds choline, and import of methylated basic amino acids via SLC25A45 can remodel mitochondrial lipid metabolism, indicating that impaired choline and methylated amino acid transport have direct effects on the efficiency of mitochondrial protein synthesis.

## RESULTS

### Members of the SLC25 mitochondrial carrier family are required for mitochondrial protein synthesis and OXPHOS stability

**Fig. 1.**
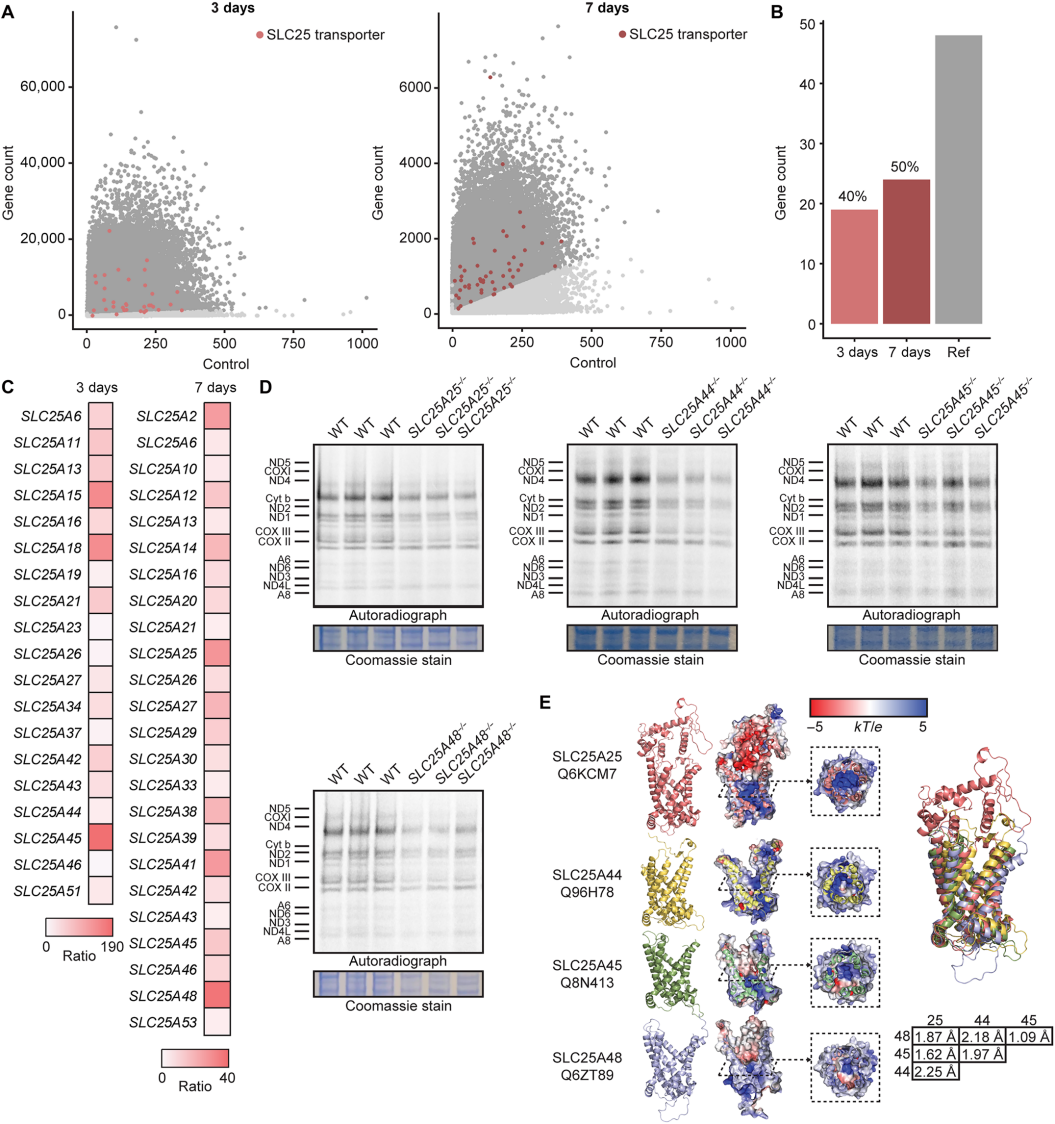
Whole-genome screen identifies SLC25 carriers that affect mitochondrial translation. (**A**) Scatterplots from a whole-genome screen of knockout genes that caused an increased GFP fluorescence of MRPL12-GFP cells in 3- and 7-day populations as an indicator of defects in mitochondrial gene expression. Guides with >3-fold enrichment relative to controls are highlighted in dark gray, and SLC25 transporters are indicated by color. (**B**) Proportions of SLC25 transporters detected by the whole-genome screen in 3- and 7-day populations after filtering for ≥3-fold enrichment, ≥2 guides, and a minimum count of 10 compared to the number present in the gRNA library. (**C**) Enrichment scores of SLC25 family members in 3- and 7-day populations determined by MaGeCK analysis compared to controls. (**D**) Mitochondrial de novo protein synthesis measured in *SLC25A25^−/−^*, *SLC25A44^−/−^*, *SLC25A45^−/−^*, and *SLC25A48^−/−^* knockout cells compared to controls [wild type (WT)] by pulse incorporation of ^35^S-labeled methionine and cysteine. Equal amounts of protein were separated by SDS-PAGE and visualized by autoradiography. (**E**) Protein structures and alignments of mitochondrial solute carrier members SLC25A25, SLC25A44, SLC25A45, and SLC25A48 obtained from AlphaFold and visualized using PyMOL.

We analyzed the enrichment of guide RNAs (gRNAs) from a whole-genome CRISPR screen using a reporter system that identifies genes affecting mitochondrial biogenesis and translation (*18*). The CRISPR library contained gRNAs targeting 48 of the 53 members of the mitochondrial solute carrier family 25 (SLC25), and we identified that 33 of these members were enriched in the screen, 3 and 7 days posttransduction (Fig. 1A). Of these, 40 and 50% were detected after gRNA count filtering in the 3- and 7-day screens, respectively (Fig. 1B), indicating that loss of these SLC25 transporters causes defects in mitochondrial biogenesis and the translation machinery. Averaged enrichment ratios between the 3- and 7-day scores compared to controls ranged from 8.1 to 181.2 at 3 days and 3.9 to 36.7 at 7 days posttransduction (Fig. 1C). gRNAs targeting *SLC25A45* had the highest enrichment score after 3 days, whereas gRNAs that targeted *SLC25A48* had the highest enrichment after 7 days.

To validate the screen results and the involvement of these transporters in mitochondrial protein synthesis, we used CRISPR-Cas9 editing to delete *SLC25A45* and *SLC25A48* in human diploid CAL51 breast cancer cells along with additional SLC25 transporters that were identified in the screen. To identify the most effective gRNAs for deleting each of the *SLC25* genes, we used two different sets of unique gRNA sequences (we dubbed each different gRNA of these pairs against the same gene as gRNA-A and gRNA-B) and measured their effects on de novo mitochondrial protein synthesis. We identified that gRNA-A against each of the *SLC25A25*, *SLC25A44*, and *SLC25A48* genes and gRNA-B against the *SLC25A45* gene significantly reduced the rate of mitochondrial translation compared to control cells for these gRNA sets (fig. S1, A and B), with corresponding effects on the formation of the OXPHOS complexes determined by blue native polyacrylamide gel electrophoresis (BN-PAGE) (fig. S1, C and D). The other sets of gRNAs were not effective at knocking out the *SLC25* genes. The effects of the successful gRNAs, which deleted each of the *SLC25* genes, on the rate of translation and the stability of OXPHOS complexes were consistent with the enrichment values identified in our screen for each gRNA, suggesting that this was a consequence of the relative importance of their target gene in supporting mitochondrial translation. We identified the greatest reduction in mitochondrial translation and OXPHOS levels upon deletion of *SLC25A45* and *SLC25A48* (fig. S1), consistent with their highest enrichments score in the screen (Fig. 1C). In addition, noticeable reduction in mitochondrial translation was found upon deletion of *SLC25A25* that encodes a mitochondrial ATP-Mg^2+^/phosphate carrier (*19*–*22*) and deletion of SLC25A44, which has been suggested to transport branched-chain amino acids into mitochondria (*23*). Recently, SLC25A48 was identified as a transporter of choline (*24*–*26*), and SLC25A45 was identified as a transporter of methylated arginine and lysine (*27*, *28*).

To understand the roles of SLC25A45, SLC25A48, SLC25A44, and SLC25A25 in mitochondrial protein synthesis, we used the gRNAs identified in our screen that effectively knocked out these *SLC25* genes. We established stable knockout human cell lines for each of these genes and validated the deletions in each cell line by Sanger sequencing (fig. S2A). We measured de novo translation in the knockout cell lines and confirmed that mitochondrial translation was significantly reduced in all of them compared to control cells (Fig. 1D and fig. S2B). Decreased mitochondrial protein synthesis is an immediate consequence of carrier loss as transient small interfering RNA (siRNA) knockdown, which rapidly reduced the levels of SLC25A48, also decreased the rate of translation (fig. S2, C and D). Analysis of AlphaFold predicted tertiary structures show that the SLC25A25, SLC25A44, SLC25A45, and SLC25A48 transporters have a threefold pseudosymmetry that is typical of the SLC25 family of proteins with a distinct central cavity (Fig. 1E) (*29*, *30*). Alignments of the predicted protein structures show that all four proteins were similar [root mean square deviation (RMSD) < 2.25 Å], which corroborates the high homology detected from multiple sequence alignments of the SLC25 family of carriers (*7*, *31*, *32*).

### Mitochondrial trans.porters are important for cell growth, OXPHOS, and mitochondrial morphology

**Fig. 2.**
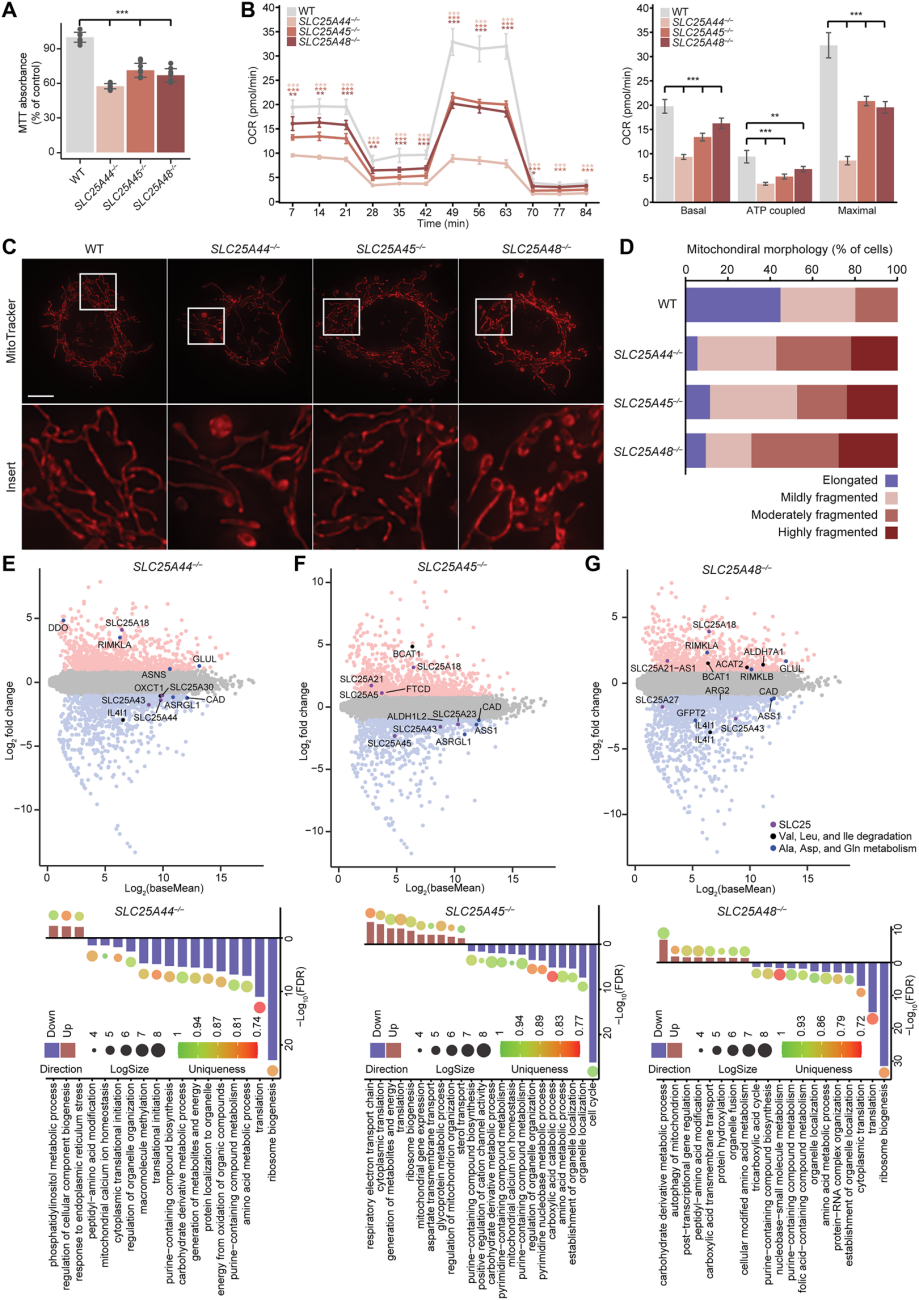
Loss of SLC25 carriers causes changes in mitochondrial function and morphology. (**A**) Changes in cellular proliferation measured using the MTT assay in control (WT), *SLC25A44^−/−^*, *SLC25A45^−/−^*, and *SLC25A48^−/−^* cells. Data are mean values ± SD of *n* = 3 biological replicates. ****P < 0.0001, a one-way ANOVA with Tukey post hoc analysis for multiple comparisons. (**B**) Oxygen consumption rate (OCR) measured using the Seahorse XF Cell Mito Stress Test kit for control (WT), *SLC25A44^−/−^*, *SLC25A45^−/−^*, and *SLC25A48^−/−^* cells. Basal, ATP-coupled, and maximal (uncoupled) respiration rates are shown. (**C**) Mitochondrial morphology of knockout cell lines compared to controls using MitoTracker Orange and visualized by fluorescence microscopy. Inserts are representative of white rectangle encompassed areas. Scale bar, 10 μm. (**D**) Mitochondrial qualitative scoring (*n* > 50) of morphology images for all cells. Details of morphology classifications are described in Materials and Methods. (**E** to **G**) Differential gene expression was measured in *SLC25A44^−/−^* (E), *SLC25A45^−/−^* (F), and *SLC25A48^−/−^* (G) cells expressed relative to control cells. Total numbers of significantly increasing and decreasing (adjusted *P* value < 0.05 and absolute log_2_ fold change > 0.5) transcript changes are shown in red and blue, respectively, and changes in specific transport genes are shown in pink, purple, and dark blue in the volcano plots along with the gene ontologies of the most significantly affected pathways.

Cell growth of the *SLC25A25^−/−^*, *SLC25A44^−/−^*, *SLC25A45^−/−^*, and *SLC25A48^−/−^* cells was significantly decreased compared to controls (Fig. 2A and fig. S3A). This was likely a consequence of the significantly reduced mitochondrial oxygen consumption in all the knockout cells, in the nonphosphorylated, phosphorylated, and uncoupled state compared to control cells (Fig. 2B and fig. S3C). Reduction in OXPHOS activity in the knockout cell lines is consistent with the reduced rate of de novo translation and biogenesis of the OXPHOS complexes, indicating that their transport activities are closely linked to energy production. Loss of each of the SLC25A25, SLC25A44, SLC25A45, and SLC25A48 transporters resulted in altered mitochondrial morphology and decreased reticular networks, compared to controls (Fig. 2, C and D, and fig. S3D). The *SLC25A25^−/−^*, *SLC25A44^−/−^*, and *SLC25A48^−/−^* cells contained the most fragmented and punctate mitochondria with severe granule mitochondrial bodies and perinuclear clustering in the *SLC25A25^−/−^* and *SLC25A48^−/−^* cells, whereas the mitochondrial network was less disrupted in the *SLC25A45^−/−^* cells.

Transcriptomic profiling revealed significant gene expression changes in each of the knockout cell lines compared to control cells (Fig. 2, E to G, and table S1). The gene expression changes in the *SLC25A44^−/−^* cells affected mitochondrial biogenesis, translation, and amino acid metabolism (Fig. 2E and table S1), and these were similar to the changes identified in the *SLC25A45^−/−^* cells (Fig. 2F and table S1). In the *SLC25A48^−/−^* cells, the most distinct changes compared to the other three knockout cells lines were related to carboxylic acid transport and folate metabolism (Fig. 2G and table S1). Altered expression of genes involved in mitochondrial translation, biogenesis, and amino acid metabolism were common features in all the knockout cells.

**Fig. 3.**
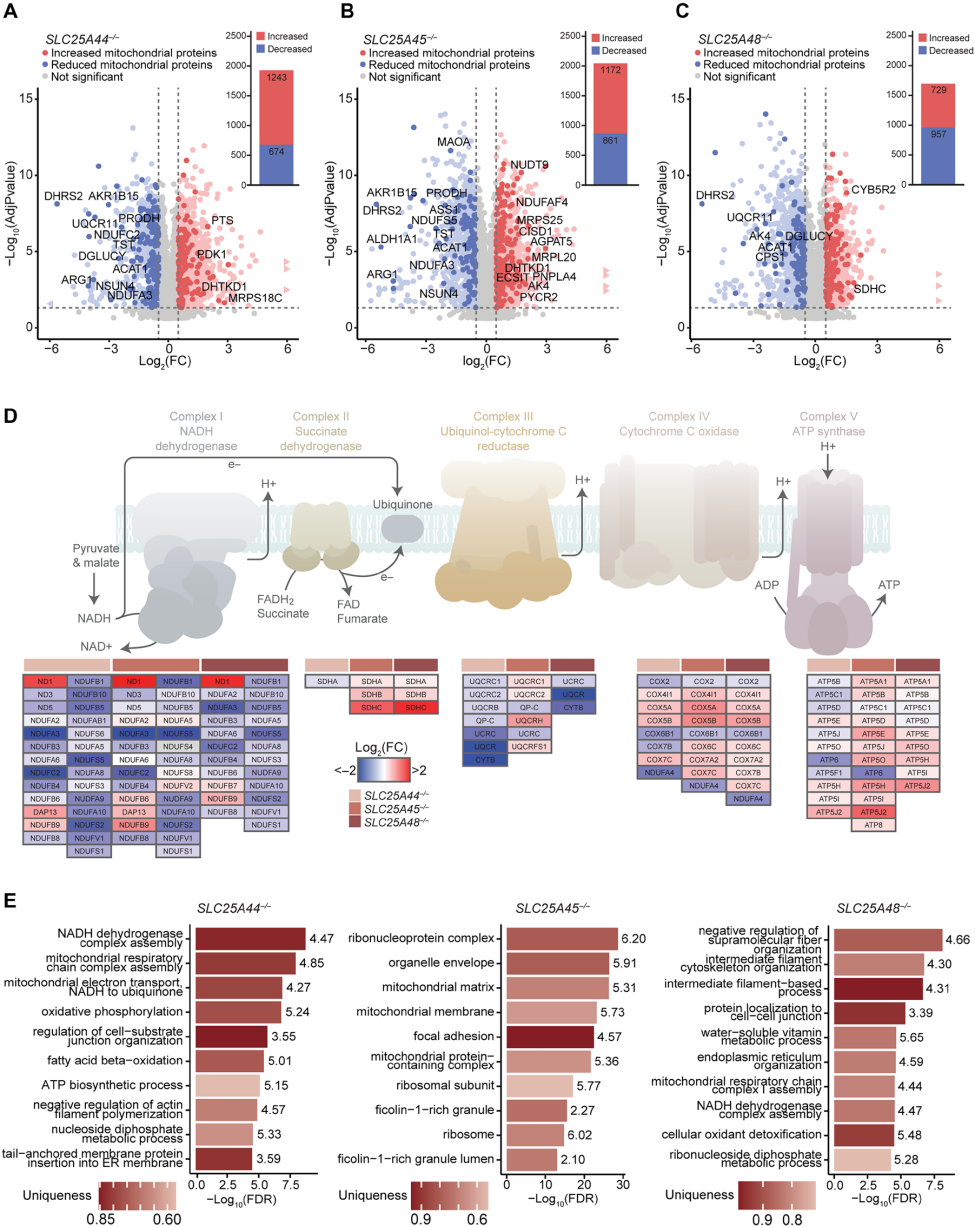
Loss of SLC25 transporters causes changes in protein abundance and affects subunits of the OXPHOS complexes. Proteomic analyses and total numbers of significantly increasing and decreasing (adjusted *P* value < 0.05 and absolute log_2_ fold change > 0.5) proteomic changes in *SLC25A44^−/−^* (**A**), *SLC25A45^−/−^* (**B**), and *SLC25A48^−/−^* (**C**) cells compared to controls shown as volcano plots. Significantly changing proteins (adjusted *P* value < 0.05) with absolute log_2_ fold change > 0.5 are indicated in light blue and red, and the significantly changing mitochondrial proteins are indicated in dark blue and red. Mitochondrial proteins involved in metabolism, OXPHOS, or translation with absolute log_2_ fold change > 1.5 are labeled. Results for the remainder of mitochondrial categories can be found in fig. S4 (A and B). (**D**) Proteomic results (adjusted *P* value < 0.05) of the subunits of the mitochondrial OXPHOS complexes for each knockout line visualized by Pathvisio software. A diagrammatic representation of the OXPHOS subunits and the movement of electrons through the system is shown at the top, and a list of the subunit proteins is presented below their corresponding complex, colored by log_2_ fold change. (**E**) Gene ontology (GO) results for significantly changing proteins that were unique to each line (adjusted *P* value < 0.05 and absolute log_2_ fold change > 0.5) in *SLC25A44^−/−^*, *SLC25A45^−/−^*, and *SLC25A48^−/−^* cells determined by Panther software. Bar graphs represent the top 10 GO results ordered by FDR, and set sizes of each ontology are indicated to the right of each bar. The complete GO result lists can be found in table S2.

Next, we carried out proteomic profiling of each of the knockout cell lines to identify the global impact of loss of each transporter on the mitochondrial proteome (Fig. 3, A to E; fig. S3, D and E; and table S2). We identified a large number of significantly increased and decreased proteins in each of the knockout cells (Fig. 3, A to C). Mitochondrial proteins involved in metabolism, OXPHOS, and protein synthesis were most affected in all knockout cells compared to controls (Fig. 3, A to C; figs. S3, D and E, and S4, A and B; and table S2). Complex I and Complex III proteins were significantly reduced across all knockout cells (Fig. 3D and fig. S3F). However, loss of SLC25A25 caused global reduction of OXPHOS proteins that are components of all five respiratory complexes (fig. S3F) and reduced mitochondrial contacts with the endoplasmic reticulum (ER) (fig. S3G). The greatest number of reduced OXPHOS proteins were subunits of Complex I in the *SLC25A44^−/−^*, *SLC25A45^−/−^*, and *SLC25A48^−/−^* cells (Fig. 3D). Complex I assembly factors were decreased in the *SLC25A25^−/−^* cells but increased in the other knockout cells (fig. S3, E and F, and table S2), indicating a more severe defect in Complex I in the absence of SLC25A25, consistent with decreased levels of all detected OXPHOS proteins. Complex II subunits were also reduced in the *SLC25A44^−/−^* cells but increased in the *SLC25A45^−/−^* and *SLC25A48^−/−^* cells, whereas most of the detected subunits of Complex III were decreased in all knockout cell lines (Fig. 3D). Most Complex IV and V subunits were reduced in the *SLC25A44^−/−^* cells compared to the varied effects on the *SLC25A45^−/−^* and *SLC25A48^−/−^* cells; however, mitochondrially encoded subunits of these complexes were reduced in all the cell lines (Fig. 3D), consistent with reduction in their translation rates. Gene ontology analyses for each cell line compared to controls further confirmed that the most affected biological processes involved mitochondrial function, OXPHOS, and cytoskeletal organization (Fig. 3E and fig. S4, A and B), consistent with defects in mitochondrial translation and morphology.

### The mitochondrial carrier SLC25A48 binds choline

**Fig. 4.**
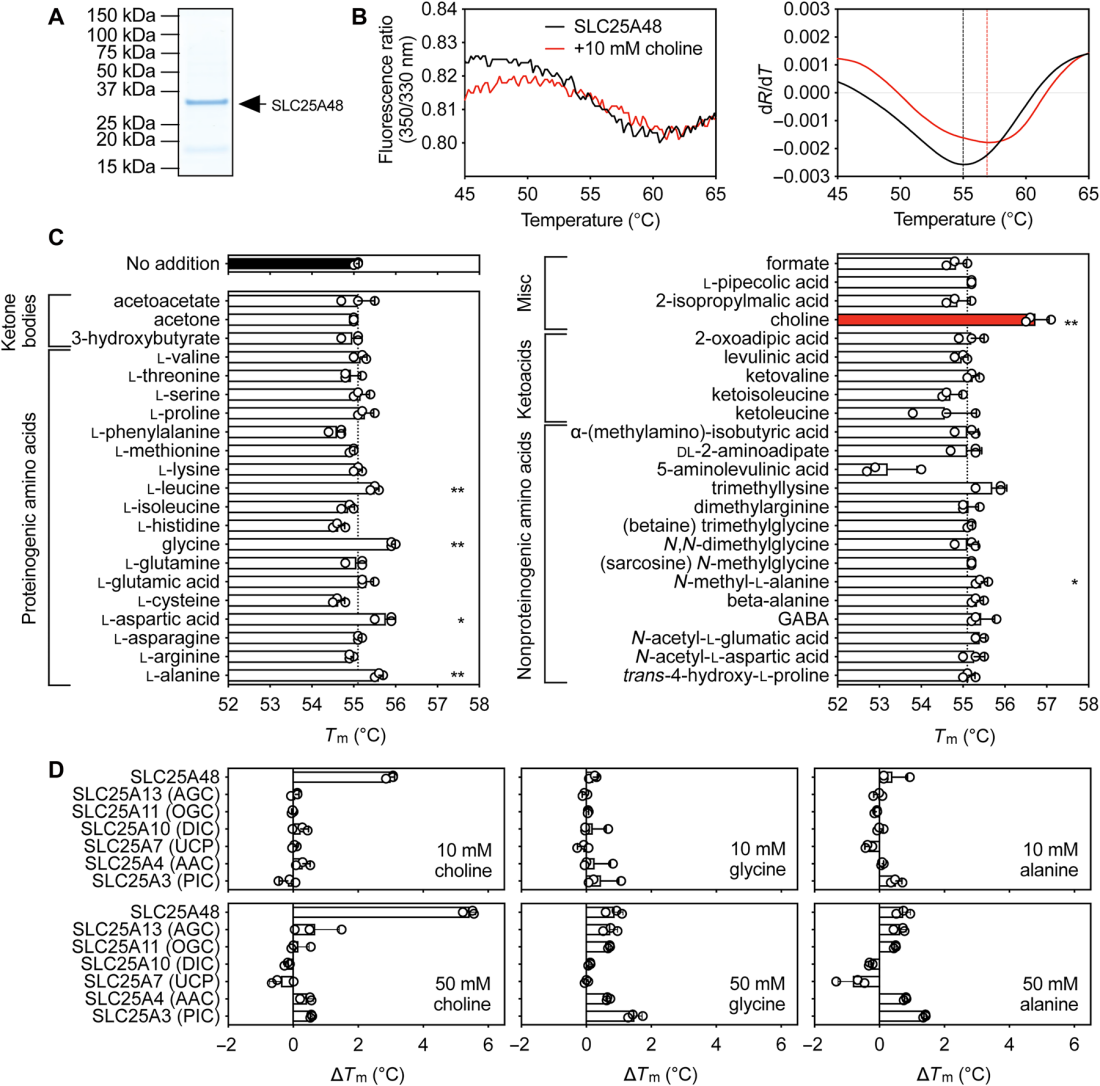
Purified human SLC25A48 is specifically stabilized by choline. (**A**) Instant-blue stained SDS-PAGE gel of purified protein (4 μg of the final sample was loaded onto the gel). (**B**) Typical unfolding curves of ~1 μg of protein without compound (black trace) or with 10 mM choline (red trace). (**C**) Apparent melting temperature of SLC25A48 in the presence of 10 mM compound. The dashed line is the apparent melting temperature of protein without compound addition. The data represent the average and SD of three biological repeats, each the average of three technical repeats. Two-tailed Student’s *t* tests assuming unequal variances were performed for the significance analysis (*P < 0.05; **P < 0.01; ***P < 0.001; ****P < 0.0001). (**D**) Change in melting temperature (∆*T*_m_), calculated by subtracting the apparent *T*_m_ in the absence of compound from the apparent *T*_m_ in the presence of compound and SLC25A3 (mitochondrial phosphate carrier), SLC25A4 (mitochondrial ADP/ATP carrier), SLC25A7 (mitochondrial uncoupling protein), SLC25A10 (mitochondrial dicarboxylate carrier), SLC25A11 (mitochondrial oxoglutarate carrier), SLC25A13 (mitochondrial aspartate/glutamate carrier), and SLC25A48 in the presence of either choline, glycine, or alanine at a concentration of 10 and 50 mM. The data represent the average and SD of three technical repeats. *P < 0.05; **P < 0.01, two-tailed Student’s *t* test.

Recently, choline has been suggested to be a substrate for SLC25A48 (*24*–*26*), and because a functional link between choline transport and mitochondrial translation was not apparent, we expressed the SLC25A48 transporter in *Saccharomyces cerevisiae* and purified it from mitochondria to survey its potential repertoire of substrates (Fig. 4A).

The apparent melting temperature (*T*_m_) at which the rate of the SLC25A48 protein unfolding is the highest was 55°C, similar to other mitochondrial carrier family proteins (Fig. 4B). We have previously shown that substrates of membrane transporters, including members of the mitochondrial carrier family, can stabilize protein in thermostability assays (*33*–*37*). We carried out a high-throughput thermal denaturation screen that included proteinogenic and nonproteinogenic amino acids, with the aim of identifying stabilizing compounds that could be additional substrates of SLC25A48. As expected, choline specifically stabilized SLC25A48 (∆*T*_m_ = 1.7°C; Fig. 4C) but not the other tested mitochondrial carriers (Fig. 4D), suggesting that choline binds specifically to SLC25A48, consistent with it being a substrate. Although glycine (∆*T*_m_ = 0.9°C) and alanine (∆*T*_m_ = 0.5°C) also stabilized SLC25A48, albeit to a lesser extent than choline, these act as nonspecific stabilizers of other SLC25 family proteins (Fig. 4D), thus are unlikely to be additional physiological substrates. The ability of SLC25A48 to act as carrier for choline was tested with transport assays, but SLC25A48 was not stable after reconstitution, and therefore these assays were not possible.

**Fig. 5.**
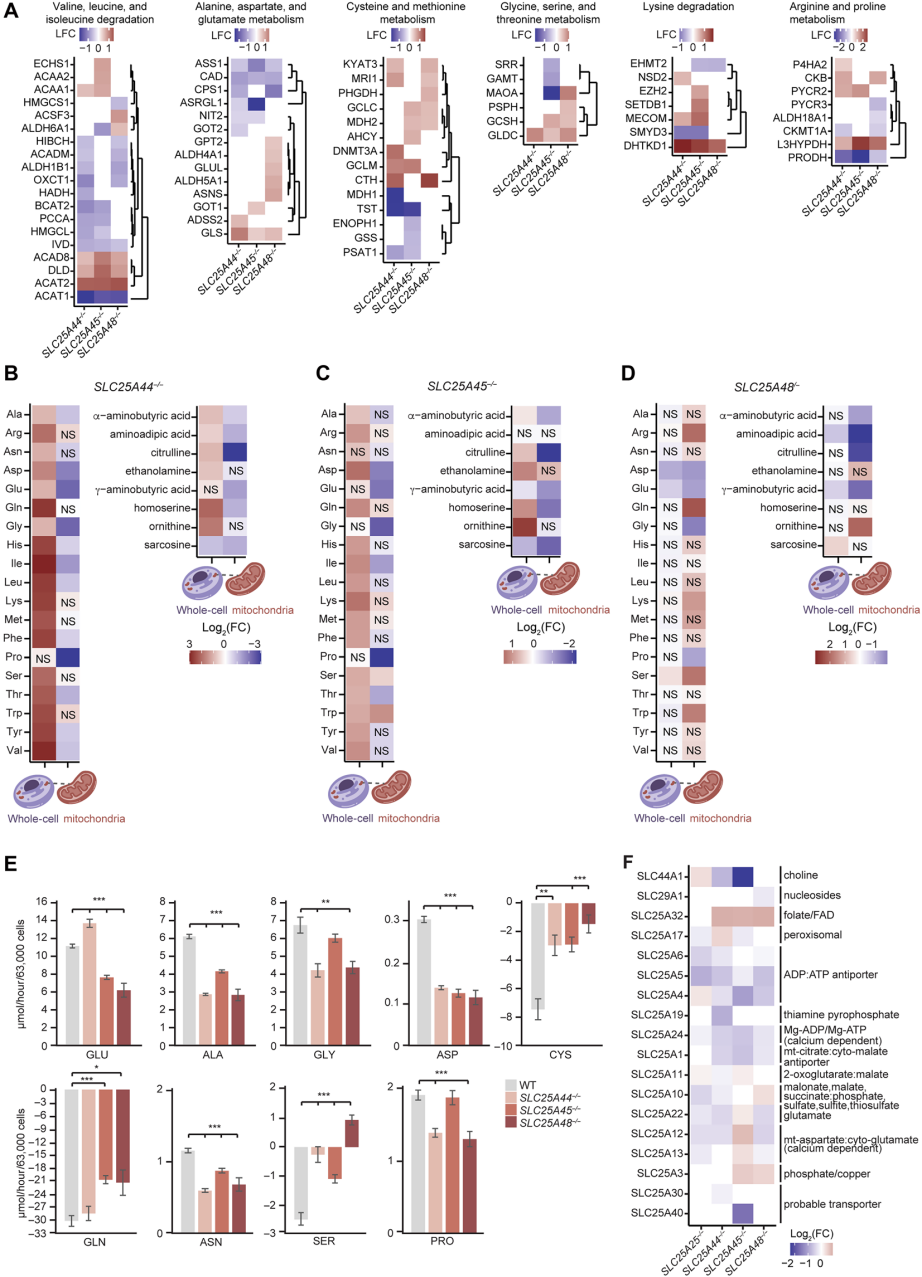
Loss of SLC25A44, SLC25A45, and SLC25A48 leads to changes in metabolic profiles and amino acid metabolism in whole cells and isolated mitochondria. (**A**) Hierarchical clustering of significantly changing (adjusted *P* value < 0.05 and absolute log_2_ fold change > 0.5) enzymes involved in amino acid metabolism detected by mass spectrometry in *SLC25A44^−/−^*, *SLC25A45^−/−^*, and *SLC25A48^−/−^* cells. Proteomic changes are summarized by amino acid metabolic processes obtained from the Kyoto Encyclopedia of Genes and Genomes (KEGG) database, with duplicates removed across different pathways. (**B** to **D**) Amino acid levels detected by mass spectrometry for *SLC25A44^−/−^* (B), *SLC25A45^−/−^* (C), and *SLC25A48^−/−^* (D) cells compared to controls in total cells and isolated mitochondria. NS indicates nonsignificant changes (significance threshold: adjusted *P* value < 0.05). (**E**) Metabolic flux analysis of metabolite levels detected in cell growth media via mass spectrometry in control (WT), *SLC25A44^−/−^*, *SLC25A45^−/−^*, and *SLC25A48^−/−^* cells. *P < 0.05; **P < 0.01; ***P < 0.001, Student’s two-sided unpaired *t* test. Error bars represent SD. (**F**) Significantly changing (adjusted *P* value < 0.05) protein levels of other SLC25 members in *SLC25A25^−/−^*, *SLC25A44^−/−^*, *SLC25A45^−/−^*, and *SLC25A48^−/−^* cells. Annotation of substrates targeted for transport for each protein is shown to the right of the graph.

Changes in amino acid levels can affect the rate of translation, and because SLC25A44 (*23*), SLC25A45 (*27*, *28*), and SLC25A48 have been directly or indirectly implicated in the transport of amino acids, we analyzed the changes in enzymes that regulate amino acid metabolism in the *SLC25A44^−/−^*, *SLC25A45^−/−^*, and *SLC25A48^−/−^* cells compared to control cells (Fig. 5A). We identified that enzymes involved in valine, leucine, and isoleucine degradation pathways were particularly affected in knockout cells, and this was most severe in *SLC25A44^−/−^* cells (Fig. 5A). These results reflect the impact of deleting SLC25A44 on enzymes involved in amino acid metabolism, particularly of branched-chain amino acids, which has been shown before (*23*). Loss of SLC25A45 affected the metabolism of lysine and arginine most significantly, consistent with the role of SLC25A45 in the import of dimethylarginine (DMA) and trimethyllysine (TML) into mitochondria (*27*, *28*) (Fig. 5A). Proteins involved in alanine, aspartate, and glutamate metabolism were particularly enriched in *SLC25A48^−/−^* cells, in addition to cysteine and methionine metabolism (Fig. 5A). The deletion of SLC25A48 affected enzymes involved in glycine amino acid metabolism consistent with the requirement of glycine as precursor for choline metabolism.

### Mitochondrial amino acid metabolism is disrupted by loss of SLC25A44, SLC25A45, and SLC25A48

Amino acids are essential for protein synthesis and because mitochondrial translation was reduced in the knockout cells, we investigated how the loss of each transporter affected amino acid metabolites in whole cells and isolated mitochondria by mass spectrometry (Fig. 5, B to D, and table S3). The total cellular pool of amino acids was significantly increased in the *SLC25A44^−/−^* cells, with the exception of proline, whereas the greater mitochondrial amino acid pool was reduced in these cells (Fig. 5B). A similar trend was identified in the *SLC25A45^−/−^* cells, with increased levels of most amino acids in the total cellular pool, and a general decrease in most amino acids within mitochondria, compared to control cells (Fig. 5C). In the *SLC25A48^−/−^* cells, only the total cellular level of serine was increased, whereas the total levels of aspartic acid and glutamic acid were significantly reduced in these cells compared to controls (Fig. 5D). In the isolated mitochondria from the *SLC25A48^−/−^* cells, glycine was the most significantly reduced amino acid, followed by proline, aspartic acid, and glutamic acid, reflecting the inability of the mitochondrion to import choline, thereby depleting glycine as a precursor for its synthesis while accumulating serine in an attempt to increase choline levels inside mitochondria (Fig. 5D). In addition to the mitochondrial serine levels, lysine, tryptophan, arginine, and glutamine levels were significantly increased as well, reflecting the interdependency of these amino acid pathways that use common substrates (Fig. 5D). We found that SLC25A44 and SLC25A45 have similar effects on amino acid metabolism (Fig. 5, B and C). The loss of SLC25A48 affected the levels of mitochondrial amino acids used by biosynthetic pathways that converge on choline metabolism (Fig. 5D), indicating that changes in the flux of mitochondrial metabolites can have imminent effects on amino acid abundance. The disruption of mitochondrial amino acid metabolism for each of the three transporters explains the reduced rate of protein synthesis and implicates the regulatory roles of these amino acid and choline transporters for mitochondrial translation.

We also measured nonproteinogenic amino acids in whole cells and isolated mitochondria. Accumulation of ornithine and citrulline in the *SLC25A44^−/−^* and *SLC25A45^−/−^* cells and their reduced mitochondrial levels (Fig. 5, B and C) suggested a buildup of urea cycle intermediates (Fig. 5, B and C), whereas a specific increase in cytoplasmic sarcosine in the *SLC25A48^−/−^* cells (Fig. 5D) indicated a buildup of one-carbon metabolites as a consequence of impaired choline import. The reduction of both the cellular and mitochondrial levels of γ-aminobutyric acid (GABA) and aminoadipic acid in the *SLC25A48^−/−^* cells (Fig. 5D) may be a consequence of reduced glutamate or aspartate levels, which may indicate impaired import of metabolites dependent on glutamate and aspartate in mitochondria.

Next, we measured the cell-specific uptake and secretion rates of each amino acid in the growth media of the knockout cells. All three *SLC25A44^−/−^*, *SLC25A45^−/−^*, and *SLC25A48^−/−^* cell lines shared common impaired amino acid catabolism profiles, with reductions in both the net amount of carbon uptake and conversion to non–amino acid products. This was most severe in *SLC25A45^−/−^* and *SLC25A48^−/−^* cell lines, with amino acid C-mole uptake rates of only 66 and 63% of control cell levels, respectively, and catabolism to non–amino acid products at 39 and 52% of control cell levels, respectively. Although some patterns in amino acid use and production were common across all three *SLC25A44^−/−^*, *SLC25A45^−/−^*, and *SLC25A48^−/−^* cell lines, cell line–specific perturbations were also observed (Fig. 5E and table S3). The excretion of alanine, aspartic acid, and asparagine was reduced in all three cell lines, whereas the excretion of glycine and proline was specifically reduced in the *SLC25A44^−/−^* and *SLC25A48^−/−^* cells. Excretion of glutamic acid was increased in the *SLC25A44^−/−^* cells only, but it was decreased in the *SLC25A45^−/−^* and *SLC25A48^−/−^* cells, suggesting that requirements for glutamic acid may be increased in these cell lines, consistent with the reduction in glutamine uptake rates in these cells. Cysteine and serine absorption was also reduced in the knockout cell lines, but serine excretion was observed in the *SLC25A48^−/−^* cells specifically (Fig. 5E), suggesting the need for this cell line to sink accumulated one-carbon metabolites. These findings suggest that disrupted import of specific amino acids into mitochondria can have complex effects on the steady-state levels of other amino acids and metabolites, as well as their flux/uptake and secretion.

We investigated the levels of other mitochondrial transporters in the knockout cells to gain insight into potential coregulation of the SLC25A44, SLC25A45, and SLC25A48 carriers with transporters whose cargos have been previously established (Fig. 5F and fig. S5A). At a transcriptomic level, the greatest significant changes were found in all knockout cell lines in the two previously uncharacterized SLC25A43 and SLC25A18 carriers, whereas the most significant specific changes were related to the loss of each of the *SLC25A44*, *SLC25A45*, and *SLC25A48* knocked out genes (fig. S5A). Loss of *SLC25A44* did not change the expression of *SLC25A45* or *SLC25A48*, and this was the case for the other two knockout cell lines, reflecting the distinct roles and substrates of each of these carriers and their independent regulation (fig. S5A). At the protein level, most of the SLC25 member proteins were coregulated in more than one of our knockout cell lines, such as the ADP/ATP carriers SLC25A4, SLC25A5, and SLC25A6, the Mg-ATP/phosphate carrier SLC25A24, and the citrate carrier SLC25A1, which were decreased in the *SLC25A44^−/−^*, *SLC25A45^−/−^*, and *SLC25A48^−/−^* cells (Fig. 5F), reflective of reduced overall OXPHOS function in these knockout cells. However, there was a unique increase in the glutamate or aspartate/glutamate transporters: SLC25A22, SLC25A12, and SLC25A13 in the *SLC25A45^−/−^* cells (Fig. 5F), suggesting a potential role for SLC25A45 in the urea cycle and arginine biosynthesis by transporting methylated arginine and lysine. In addition, there was an increase in the dicarboxylate carrier SLC25A10 (*33*, *34*) specifically in the *SLC25A48^−/−^* cells, pointing to the increased requirement of succinate for OXPHOS and redox functions or for heme biosynthesis as glycine, an amino acid that is required for both of these pathways, levels were severely reduced in the mitochondria of the *SLC25A48^−/−^* cells. Increase in neutral plasma transporters suggested that it may be a compensatory response to impaired amino acid import into mitochondria as increased levels of amino acids in the cytoplasm cannot correct the mitochondrial defect (fig. S5B).

### Lipidome remodeling in SLC25 knockout cells

**Fig. 6.**
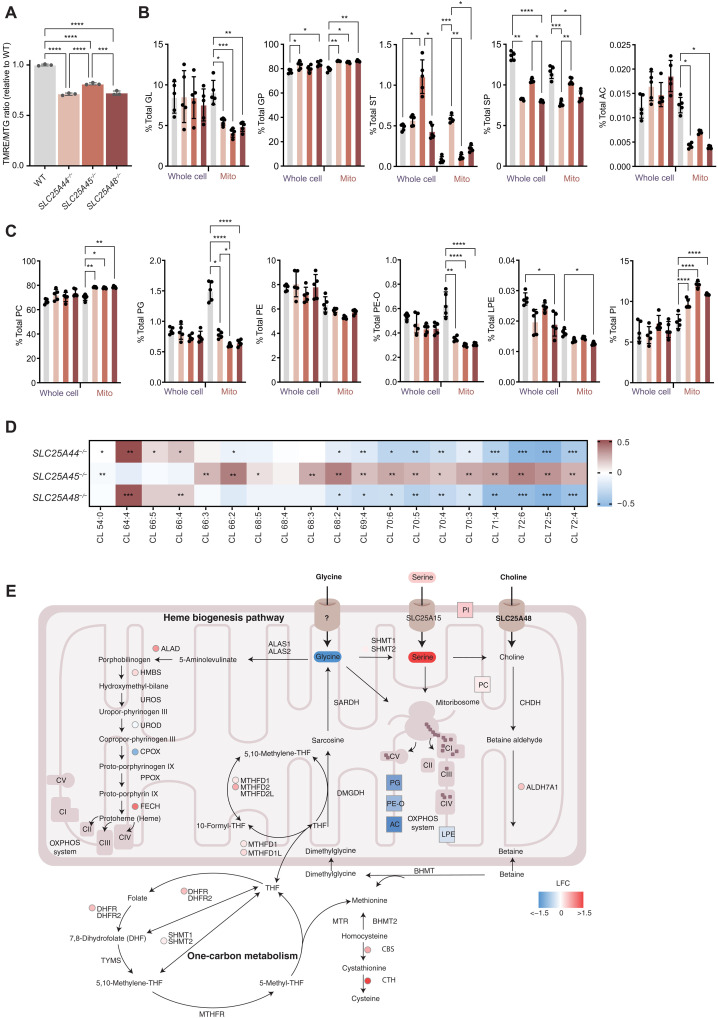
The mitochondrial carrier SLC25A48 regulates mitochondrial protein synthesis and the heme biosynthetic pathway via import of glycine, alanine, and choline. (**A**) TMRE was used to measure ΔΨ_m_, and data were normalized to mitochondrial content using the ΔΨ_m_ -insensitive dye MTG, showing the TMRE/MTG ratio. Data are mean values ± SD of *n* = 3 biological replicates. ***P < 0.001; ****P < 0.0001, a one-way ANOVA with Tukey post hoc analysis for multiple comparisons. (**B**) Lipid levels in control and knockout cells determined by mass spectrometry in *n* = 5 (WT, *SLC25A44^−/−^*, *SLC25A45^−/−^*, and *SLC25A48^−/−^* cells). The abundances of glycerophospholipids (GP), glycerolipids (GL), sterols (ST), and sphingolipids (SP) and acylcarnitines (AC). (**C**) Specific lipid classes are shown: phosphatidylcholine (PC), phosphatidylglycerol (PG), phosphatidylethanolamine (PE), monoalkyl phosphatidylethanolamine (PE-O), lysophosphatidylethanolamine (LPE), and phosphatidylinositol (PI). (**D**) Different acyl chain compositions of cardiolipin are shown as a heatmap. Data are calculated as % of control abundance. FDR < 0.05, *; FDR < 0.01, **; FDR < 0.001, ***. (**E**) Significantly changing proteomic, metabolomic, and lipidomic (adjusted *P* value < 0.05) changes mapped onto mitochondrial pathways in the *SLC25A48^−/−^* cells.

Mitochondrial translation in mammalian cells is dependent on the membrane potential across the inner membrane (*14*), and this was evident from the reduced membrane potential in the knockout cells compared to control cells (Fig. 6A and fig. S3B), which correlated with their reduced rate of translation. Mitochondria and ER contact sites, required for cytoplasmic ribosomes to be in close proximity to mitochondria to coordinate the cotranslational insertion of mitochondrial proteins (*38*), were significantly reduced in all of the knockout lines consistent with mitochondrial fragmentation (fig. S6, A and B). In addition, changes in the mitochondrial membrane potential have been associated with phospholipid remodeling (*39*) and reduced ether lipid metabolism has been identified to cause mitochondrial fragmentation and swelling as a consequence of impaired organelle biogenesis (*18*). Therefore, we profiled the cellular and mitochondrial lipidomes in the knockout and control cells, identifying a general reduction in total mitochondrial glycerolipid (GL) levels with a concomitant increase in their glycerophospholipid (GP) and sterol (ST) levels (Fig. 6B and table S4). The total sphingolipid (SP) and mitochondrial SP levels followed a similar reduced trend in the knockout compared to control cells (Fig. 6B). The mitochondrial acylcarnitine (AC) levels were significantly reduced in all three knockout cell lines relative to controls (Fig. 6B), suggesting that the reduction in AC may contribute to the reduced membrane potential and mitochondrial fragmentation. Phosphatidylcholine (PC) lipids in mitochondria, which are the most abundant GP, were increased and followed the same trend as the total GP levels (Fig. 6C), whereas mitochondrial phosphatidylglycerol (PG) levels, required for cardiolipin (CL) synthesis, were significantly reduced in all of the knockout cell lines. These along with the AC levels are indicative of mitochondrial dysfunction and impaired dynamics. Although mitochondrial phosphatidylethanolamine (PE) levels were not changed, the ether-linked phosphatidylethanolamine (PE-O) levels, which reside in the mitochondrial inner membrane and provide the fluidity of the membrane (*40*) much like PG and AC, were significantly reduced in the knockout cells (Fig. 6C), further confirming reduction in the fluidity of the mitochondrial membrane. We identified a specific reduction in the total and mitochondrial lysophosphatidylethanolamine (LPE) levels in the *SLC25A48^−/−^* cells and an increase in phosphatidylinositol (PI) levels in the mitochondria of the *SLC25A48^−/−^* cells (Fig. 6C). We found that loss of either SLC25A44 or SLC25A48 led to reduced levels of mature CL species, whereas loss of SLC25A45 caused an accumulation of longer and more unsaturated CL species, suggesting up-regulation of CL biosynthesis and remodeling (Fig. 6D). Transcriptome and proteome analyses of GP enzymes identified significant reduction in the transcript and protein levels of STARD7, a protein known to transport PC into mitochondria, in the *SLC25A25^−/−^*, *SLC25A44^−/−^*, and *SLC25A48^−/−^* cells but not in the *SLC25A45^−/−^* cells, correlating with remodeling of the mitochondrial GP profile and, most notably, reduction in PG and CL levels (fig. S5, C and D). Furthermore, we found that there are common transcriptional and proteomic changes in phosphoglycerolipid enzymes related to STARD7 across the knockout cell lines (fig. S5C).

### SLC25A48 modulates mitochondrial translation via heme biosynthesis and one-carbon metabolism

An integrated analysis of our proteomic, metabolomic and lipidomic data revealed that loss of SLC25A44, SLC25A45, and SLC25A48 caused reduction in mitochondrial protein synthesis by several means. The loss of SLC25A44 led to reduced amino acids required for mitochondrial protein synthesis (Fig. 5B and fig. S7A), whereas impaired import of the carnitine biosynthesis substrates DMA and TML in mitochondria resulted in decreased acyl carnitine levels, shifting lipid metabolism toward increased GP and CL biosynthesis as an alternative storage or buffering mechanism for excess cytoplasmic acyl chains (Fig. 6, B to D, and fig. S7B). Increasing concentrations of carnitine were able to marginally but significantly improve the cell growth of the *SLC25A45^−/−^* cells (fig. S7C), further validating the role of this transporter in methylated amino acid transport (*27*, *28*). The reduced glycine levels in the *SLC25A48^−/−^* cells directly affected the rate of mitochondrial protein synthesis, causing a reduction in membrane potential (Fig. 6A), as a consequence of reduced AC, PG, PE-O, and LPE levels that impair the fluidity of the inner mitochondrial membrane, resulting in decreased OXPHOS stability and consequently oxygen consumption (Figs. 2B and 3D). Reduction in glycine likely further compounds OXPHOS biogenesis defects as it is a substrate for the initial steps of the heme biosynthesis, which is required for the stability of numerous subunits of the respiratory chain. This two-pronged disruption of OXPHOS complexes can, in turn, lead to up-regulation of both mitochondrial and cytoplasmic one-carbon metabolism enzymes, as a stress response to mitochondrial dysfunction (Fig. 6E), where increased concentrations of exogenously supplemented betaine can improve but not fully recover the cell growth and mitochondrial translation of the *SLC25A48^−/−^* cells (fig. S7, D and E).

## DISCUSSION

Mitochondria act as key regulators of cellular metabolism as they are the sites for the biosynthesis of a wide variety of macromolecules. However, the impact of cellular metabolic processes on mitochondrial translation is poorly understood. Mitochondrial gene expression and cellular metabolism have been shown to be intrinsically linked (*18*, *41*, *42*). Proteins involved in mitoribosome formation, accessory translation factors, and RNA modifications are required for dynamic reprogramming of metabolism and their loss can lead to metabolic defects (*17*, *41*, *43*). In these instances, up-regulation of mitochondrial translation has been the driver of metabolic remodeling, whereas impaired mitochondrial protein synthesis causes mitochondrial dysfunction and multisystemic diseases (*17*, *41*, *43*). Mitochondrial SLC25 transporters function at the interface of mitochondrial and cytoplasmic metabolism, shuttling essential amino acids, metabolites, and ions in and out of the mitochondrial matrix, and are required for metabolic homeostasis (*44*). Here, we show that many members of the SLC25 family play important roles in mitochondrial translation as they were enriched in our screen for mitochondrial biogenesis regulators (*18*). Most of the identified SLC25 carriers were those that transport amino acids, which is consistent with significant reduction of amino acids and consequent direct effects on mitochondrial translation rates and OXPHOS. The SLC25A48 transporter was identified to play an important role in choline balance in mitochondria and cells (*24*–*26*). Here, we validated that choline binds specifically to SLC25A48. This observation has important implications for mitochondrial functional regulation, brown fat thermogenesis (*24*), as well for genetic variants (*26*) and loss of function mutations (*45*) that are found in the human population. Recently, SLC25A45 was identified as a transporter of DMA and TML, which are precursors required for de novo carnitine synthesis (*27*, *28*). SLC25A45 has been found to be coregulated with SLC25A44, a transporter of branched-chain amino acids (*23*). SLC25A44 and SLC25A45 have complementary tissue expression profiles; SLC25A44 is highly expressed in the brain, skeletal muscle, brown adipose tissue (*23*), liver, adipose tissue, and parts of the central nervous system (*46*), whereas SLC25A45 is expressed in specific regions of the brain, skeletal muscle, intestines (*46*), and pancreas (*47*), suggesting tissue-specific requirements for transport of their amino acid substrates. We show that SLC25A45 loss caused reduction in mitochondrial acylcarnitine levels, as a consequence of impaired import of their substrates DMA and TML consistent with recent findings (*27*, *28*), which we show led to compensatory accumulation of CL and remodeling of mitochondrial lipids. This expands our knowledge about the effects of these transporters on amino acid and lipid metabolism that can affect mitochondrial protein synthesis.

Our integrative analysis reveals that SLC25A48 plays a critical and previously unappreciated role in maintaining mitochondrial lipid homeostasis and bioenergetics, likely through its involvement in choline import. Loss of SLC25A48 altered mitochondrial lipid composition, accompanied by impaired OXPHOS, loss of mitochondrial membrane potential, and reduced mitochondria-ER contacts, which was consistent with fragmentation of the mitochondrial network, suggesting that SLC25A48 is essential for coupling mitochondrial membrane composition with metabolic flux and structural integrity. Increased PC levels in the mitochondria of the *SLC25A48^−/−^* cells were likely compensatory in response to disrupted import of choline or its intermediates, which are required for de novo PC synthesis via the Kennedy pathway and for PE methylation (*48*, *49*).

Mitochondrial LPE is primarily generated through the phospholipase A–mediated turnover of PE and is tightly regulated to preserve mitochondrial membrane curvature and dynamics (*50*). Despite unaltered PE levels, the observed LPE depletion suggests a disruption in mitochondrial membrane remodeling or phospholipid salvage pathways, potentially due to impaired methylation flux. Given that choline-derived betaine is a cytosolic, folate-independent one-carbon donor, and its products, such as dimethylglycine and sarcosine, are mitochondrial folate-dependent one-carbon donors (*51*), loss of SLC25A48 likely attenuates intramitochondrial methylation reactions. Reduction in mitochondrial PI, whose biosynthesis also depends on cytidine diphosphate–diacylglycerol (CDP-DAG) and methylation-dependent headgroup exchange (*52*), further supports the lipid changes we identified. The altered amino acid profile in the *SLC25A48^−/−^* cells aligns with these metabolic shifts. Reductions in glycine, aspartate, glutamate, proline, citrulline, and aminoadipic acid suggest broad disturbances in central carbon metabolism, spanning the tricarboxylic acid (TCA) cycle, amino acid interconversions, and one-carbon pathways. Among these, glycine is particularly notable: In mitochondria, it is both consumed and produced through the folate cycle, where its cleavage contributes one-carbon units and supports reduced form of nicotinamide adenine dinucleotide phosphate (NADPH) generation via methylenetetrahydrofolate dehydrogenases. Depletion of glycine under conditions of choline insufficiency would therefore be expected not only to impair biosynthetic one carbon flux but also to limit mitochondrial NADPH production, potentially exacerbating redox imbalance (*53*). Conversely, increases in ornithine, arginine, lysine, serine, and glutamate may reflect compensatory activation of the urea cycle and amino acid catabolism to maintain nitrogen balance and support anaplerotic fluxes in response to mitochondrial stress. Mitochondrial fragmentation and loss of membrane potential are hallmarks of disrupted lipid homeostasis (*54*), and reduction of PI and LPE, which are essential for cristae morphology, fission/fusion dynamics, and respiratory supercomplex stability, could contribute to these defects in the *SLC25A48^−/−^* cells. The diminished OXPHOS capacity observed upon SLC25A48 loss is therefore likely a downstream consequence of this lipid and metabolite imbalance, compounding the energetic crisis initiated by defective methylation and membrane remodeling.

The significant reduction in mitochondrial translation observed in all knockout cells can be directly attributed to the integrated effects of altered lipid composition, metabolite depletion, and mitochondrial dysfunction. Mitochondrial translation requires tightly regulated membrane environments for ribosome docking, cotranslational insertion of nascent peptides, and assembly of OXPHOS complexes (*38*, *55*). These changes likely destabilize the cristae membrane domains required for mitochondrial ribosome attachment and impair the insertion of nascent polypeptides. Functionally, the knockout cells also had marked reduction in mitochondrial membrane potential, which is essential for the import of nuclear-encoded translation factors, translation, and the insertion of hydrophobic proteins into the inner membrane. These changes reduced mitochondria-ER contacts resulting in mitochondrial fragmentation in all knockout cell lines. The specific loss of the Ca^2+^-regulated ATP-Mg^2+^/P_i_ SLC25A25 carrier has been shown to disrupt mitochondrial energy and ion homeostasis (*22*, *56*) that normally helps keep the fusion-fission machinery balanced, compounding mitochondrial fragmentation. Unlike the SLC25A44/45/48 transporters, whose defects are more metabolic and indirect, SLC25A25 loss directly affects matrix ATP levels and Ca^2+^ levels (*22*, *56*), which are upstream triggers of fission, possibly resulting in the greater effects on mitochondrial fragmentation.

We provide further experimental support that SLC25A48 functions by facilitating the entry of choline into the mitochondrial matrix. We propose that this transport is necessary to sustain mitochondrial membrane potential, the rate of translation, and membrane lipid replenishment during mitochondrial biogenesis and stress adaptation. The consequences of SLC25A48 loss are not limited to isolated lipid alterations; rather, they reflect a systemic breakdown of mitochondrial metabolic coordination. The observed increases in GM3 and sterol species (table S4) also suggest broader perturbations in sterol metabolism and glycosphingolipid trafficking, processes that have been linked to mitochondria-ER contact sites (*57*), which we have shown to be reduced upon loss of SLC25A48. Furthermore, the addition of betaine in cells lacking SLC25A48 may marginally improve mitochondrial translation and cell growth via its properties as an osmolyte and protein stabilizer (*58*). Thus, SLC25A48 may also play a role in tethering or signaling beyond transport. Given the essential roles of mitochondrial lipid composition in apoptosis, calcium buffering, and organelle communication, these findings place SLC25A48 at the intersection of lipid metabolism, bioenergetics, and cell fate regulation. Understanding the precise molecular role of SLC25A48, as well as its regulation under physiological and stress conditions, will be critical for determining its relevance in diseases associated with mitochondrial dysfunction, including neurodegeneration and metabolic disorders.

## MATERIALS AND METHODS

### Genome-wide CRISPR screen data collection and analysis

A mitochondrial translational stress reporter dataset containing single guide (sgRNA) read counts (*18*) was reanalyzed to examine the potential roles of mitochondrial transporters in mitochondrial translation. This dataset contained read counts obtained from CAL51–MRPL12–green fluorescent protein (GFP) cells that were transduced with the Brunello gRNA library and were sorted by flow cytometry for fluorescence 3 and 7 days posttransduction, generating a sample collection of cells with increased fluorescence. The MaGeCK v0.5.2 (*59*) count subcommand was used on the 3- and 7-day samples to generate sgRNA read count tables with default parameters using the Brunello human gRNA library as the reference. MaGeCK read count tables were then filtered to sgRNA counts with a minimum of threefold change between experimental and control and a minimum of two detected sgRNAs for each gene. sgRNAs with less than 10 counts were also discarded. Mitochondrial solute carrier family members (SLC25) were identified in both 3- and 7-day samples.

### Cell culture

CAL51-mCherry-Cas9 cells were cultured in Dulbecco’s modified essential medium (DMEM) (Gibco, Life Technologies) containing glucose (4.5 g/liter), 2 mM glutamine, 10% v/v fetal bovine serum (FBS), 1 mM sodium pyruvate, and uridine (50 μg/ml) at 37°C in 95% humidified air with 5% CO_2_. Cells were a kind gift from V. Wickramasinghe, authenticated by short tandem repeat profiling and were confirmed to be free of mycoplasma, provided in table S5.

### Generation of transient and knockout cell lines

For transient transfections, CAL51-mCherry-Cas9 cells seeded at 60% confluency were allowed to attach overnight and were transfected with 1.5 μg of a pCLIP-dual-SFFV-ZsGreen plasmid (TransEDIT, Victorian Centre for Functional Genomics) encoding dual SLC25-targeting gRNAs using FuGENE HD (Promega) in Opti-MEM (Invitrogen, Thermo Fisher Scientific). Experiments were conducted 72 hours posttransfection. To establish clonal knockout cell lines, transfected cell populations were single cell sorted 72 hours posttransfection. On the basis of the GFP fluorescence signal using a FACSAria II, cells in phosphate-buffered saline (PBS) + 2% FBS (v/v) (BD Biosciences) were individually sorted into 96-well plates. SLC25 allele knockouts were confirmed by Sanger sequencing performed by the Australia Genomic Research Facility (AGRF).

### Preparation of cell lysates

Cells were trypsinized and resuspended in a lysis solution containing 150 mM NaCl, 0.1% Triton X-100, 50 mM tris-HCl (pH 8.0), and 1x cOmplete Protease Inhibitor Cocktail (Roche) for 30 min at 4°C. Cells were then centrifuged at 21,000*g* for 15 min at 4°C to clarify lysates. Protein concentration was determined by bicinchonic acid (BCA) assay.

### Mitochondrial isolation

Mitochondria were isolated from cells by resuspending cell pellets in ice-cold swelling buffer [10 mM NaCl, 1.5 mM MgCl_2_, and 10 mM tris-HCl (pH 7.5)] and were allowed to incubate on ice for 40 min. Cells were briefly homogenized in a glass Dounce homogenizer, and the sucrose concentration was adjusted to 250 mM with 2 M sucrose in T_10_E_20_ buffer [10 mM tris-HCl and 1 mM EDTA (pH 7.6)]; the homogenate was centrifuged at 1300*g* at 4°C for 3 min. Supernatants were collected and centrifuged at 15,000*g* at 4°C for 15 min to pellet mitochondria. Protein concentration was determined by BCA assay.

### Translation assays

Cells were seeded at 70% confluency and allowed to attach overnight. Growth medium was replaced with DMEM lacking methionine and cysteine and incubated for 30 min at 37°C. Cells were then treated with emetine (100 μg/ml; Sigma-Aldrich) and incubated for 5 min. Cells were then supplemented with 200 μCi of Express35S protein labeling mix (^35^S) (PerkinElmer) and incubated for 1 hour at 37°C. Cells were pelleted in ice-cold PBS, and protein concentration was determined by BCA assay. Twenty microgram of protein was separated on a 4 to 12% Bis-Tris gel (Invitrogen), equal loading was confirmed by Coomassie staining, and autoradiography signals were visualized on a Typhoon FLA 9500 (GE Healthcare). For de novo complex assembly, 50 μg of labeled cells was separated on a 3 to 12% Bis-Tris gel (Invitrogen) by blue native electrophoresis (BN-PAGE). Equal loading was confirmed by Coomassie staining, and autoradiography signals were visualized on a Typhoon FLA 9500 (GE Healthcare).

### Fluorescence microscopy

Cells seeded overnight on 22-mm^2^ coverslips were stained with 100 nM MitoTracker Orange (Thermo Fisher Scientific) in FBS-free DMEM, incubated for 15 min at 37°C, and subsequently fixed with 4% paraformaldehyde (w/v) in PBS for 30 min at room temperature. Cells were mounted in 1,4-diazabicyclo[2.2.2]octane/polyvinyl alcohol (DABCP/PVA) medium. Cells were washed in FBS-free DMEM before staining, fixing, and mounting. Images were acquired using a DeltaVision fluorescence microscope (GE Healthcare) with a 60x 1.58–numerical aperture (NA) oil immersion objective and CoolSNAP HQ2 CCD camera and are presented as deconvoluted maximum projections of 0.2-μm optical sections using softWoRx software (GE Healthcare). Qualitative scoring of 50 different cells from two independent slides were categorized as follows: elongated, most of the mitochondrial network exhibited an interconnected, reticular appearance; mildly fragmented, minor fragmentation of the mitochondrial network but was otherwise an interconnected, reticular appearance; moderately fragmented, minor interconnection of the mitochondrial network but otherwise exhibited a fragmented appearance; highly fragmented, most of the mitochondrial network was highly fragmented.

### RNA sequencing and differential expression analysis

RNA was isolated from cells using a miRNeasy mini kit (Qiagen) incorporating an on-column RNase-free DNase digestion. Libraries were constructed using an Illumina stranded mRNA preparation kit and sequenced by AGRF using a NovaSeq 6000 platform. Sequenced reads were trimmed with Trim Galore (v0.6.9) using cutadapt (v4.2) (--paired --fastqc). Trimmed reads were mapped against a NuMTs masked human genome sequence obtained from GENCODE (GRCh38, primary assembly) using STAR (v2.7.8a) and a human GENCODE v42 gene annotation with a customized mitochondrial annotation. Salmon (v1.3.0) was used in alignment-based mode (-l ISR --seqBias --gcBias) to quantify gene expression from the transcriptome alignments produced by STAR. Differential expression analysis was performed with DESeq2 by counts summarized with tximport. Gene ontology analysis was performed with PANTHER and summarized by REVIGO.

### Quantification of mitochondrial membrane potential by flow cytometry

Mitochondrial membrane potential (Δψ_m_) was quantified using tetramethylrhodamine ethyl ester (TMRE) staining combined with MitoTracker Green (MTG) normalization in live cells. Cells were seeded into 24-well plates (1 × 10^5^ cells per well) 24 hours before staining. TMRE (Merck, #87917) and MTG (Thermo Fisher Scientific, #H3570) stock solutions were prepared at 2 and 1 mM in dimethyl sulfoxide (DMSO), respectively, and diluted in culture media to final staining concentrations of 25 nM TMRE and 50 nM MTG. Where indicated, cells were pretreated with 20 μM carbonyl cyanide *p*-trifluoromethoxyphenylhydrazone (FCCP) in serum-containing media for 10 min at 37°C to serve as a negative control for Δψ_m_. Staining was performed for 15 min at 37°C in serum-containing media. After staining, cells were washed with PBS, harvested by trypsinization, and resuspended in PBS with 2% FBS. Cell suspensions were passed through cell strainers and kept on ice before flow cytometry. Analysis was performed using a BD LSRFortessa instrument equipped with PE (TMRE) and FITC (MTG) detection channels. Compensation controls were prepared using single-stained samples, and data were exported as FCS files for downstream analysis. Fluorescence intensities of TMRE and MTG were quantified from singlet populations using FlowJo (BD) or custom scripts in R. The Δψ_m_ of each sample was determined by calculating the ratio of geometric mean fluorescence intensities of TMRE to MTG (TMRE/MTG). Group comparisons were performed using a one-way analysis of variance (ANOVA) with Tukey post hoc analysis for multiple comparisons, and Δψ_m_ values were presented relative to control conditions.

### Respiratory chain function and complex activity

Mitochondrial oxygen consumption rate was measured by seeding 35,000 cells left to attach overnight. Cells were washed with XF Seahorse assay medium (Dulbecco’s modified Eagle’s medium Seahorse XF assay media supplemented with 10 mM glucose, 2 mM l-glutamine, and 1 mM sodium pyruvate) and incubated in XF Seahorse assay medium at 37°C without CO_2_. Oxygen consumption rate was then measured using a Seahorse XF analyzer (Agilent) with the Seahorse XF Cell Mito Stress Test kit (Agilent) using 2.5 μM oligomycin, 2 μM FCCP, and 0.5 μM rotenone/antimycin. Individual well measurements were normalized by cell number.

### Flow cytometry measurements of organelle contacts using SPLICS

The indicated cell lines were transfected with plasmids for measuring interactions between mitochondria-ER as described previously (*60*) and an mCherry-encoding plasmid in a 9:1 ratio as described above and incubated for 72 hours. Cells were trypsinized, and the fluorescence signal of GFP and mCherry was measured in PBS + 2% FBS (v/v) using a BD LSRFortessa. Data are expressed as a ratio of the geometric mean of GFP:mCherry intensity for double-positive cells.

### Cell proliferation

Cells were seeded and allowed to attach overnight. Twenty microliters of CellTiter 96 Aqueous One Solution Reagent (Promega) was added to each well, and cells were incubated under standard grown conditions for 2 hours. For treatments, cells were incubated with increasing concentrations of carnitine or betaine for 72 hours as indicated in the figure legends. Absorbance was detected at 490 nm via a CLARIOstar Plus plate reader (BMG Labtech).

### Preparation of protein digests for proteomics analysis

Cells were lysed in lysis buffer (5% SDS and 50 mM triethylammonium bicarbonate), and 200 μg of protein was dissolved in 50 μl of cell lysis buffer and digested using S-trap Micro Spin columns (ProtiFi) as per the manufacturer’s instructions. Briefly, dithiothreitol was added to a final concentration of 20 mM and incubated at 70°C for 60 min. Proteins were alkylated by adding iodoacetamide to a final concentration of 40 mM and incubating at room temperature in the dark for 30 min. Proteins were acidified with 2.5 μl of 12% phosphoric acid and diluted with 165 μl of binding buffer (90% methanol and 100 mM final Tris). Samples were added to the S-Trap Micro Spin columns by centrifugation at 4000*g* for 30 s and then subsequently washed three times by successively loading 150 μl of binding buffer and centrifuging at 4000*g* for 30 s. Digestion was achieved by adding 1 μg of sequencing-grade trypsin (Promega) and 25 μl of 50 mM ammonium bicarbonate and incubating overnight at 37°C. Peptides were eluted by successively adding 40 μl of 5% acetonitrile in 0.1% formic acid, 40 μl of 50% acetonitrile in 0.1% aqueous formic acid, and 40 μl of 75% acetonitrile in 0.1% formic acid with a 30-s centrifugation step at 4000*g* between the addition of each elution buffer. The eluants were pooled, dried in a vacuum centrifuge, and resuspended in 20 μl of buffer A (5% acetonitrile in 0.1% formic acid).

### Proteomics liquid chromatography and mass spectrometry

Samples were analyzed using a Thermo Fisher Scientific Ultimate 3000 RSLC UHPLC and a Q Exactive HF mass spectrometer (Thermo Fisher Scientific). Samples were injected on a reversed-phase PepMap 100 C18 trap column (5 μm, 100 Å, 150-μm internal diameter by 5 mm) at a flow rate of 15 μl/min. After 3 min, the trap column was switched in-line with a Waters nanoEase M/Z Peptide CSH C18 resolving column (1.7 μm, 130 Å, 150-μm internal diameter by 100 mm) and the peptides were eluted at a flow rate of 3 μl/min buffer A (0.1% aqueous formic acid) and buffer B (80% acetonitrile in 0.08% aqueous formic acid) as the mobile phases. The gradient consisted of the following: 8% B for 0 to 3 min, 8 to 10% B from 3 to 6 min, 10 to 24% B from 6 to 43 min, 24 to 40% B from 43 to 51 min, and 40 to 95% B from 51 to 57 min. After 1 min at 95% B, the solvents are returned to 8% buffer B and the column is equilibrated before the next injection.

The mass spectra were obtained in data-independent acquisition (DIA) mode with an MS1 resolution of 120,000, automatic gain control target of 3 × 10^6^, maximum injection time at automatic settings, and scan range from 400 to 1100 mass/charge ratio (*m/z*). DIA spectra were recorded at a resolution of 30,000 and an automatic gain control target of 2 × 10^5^. The 70 isolation windows were 10 *m/z* each from mass 405 to 1095.

### DIA mass spectrometry data analysis

Data analysis was performed with Spectronaut version 15 (15.0.210615.50606) using direct DIA analysis and default settings unless otherwise specified (*61*). Briefly, spectra were searched against either the *Homo sapiens* proteome database, downloaded from UniProt 13/03/2020, containing 74,811 sequences. Carbamidomethylation was set as a fixed modification, and methionine oxidation and N-terminal acetylation were set as variable modifications with 1% false discovery rate (FDR) cutoffs at the peptide spectral match, peptide, and protein group levels. Quantitation was performed at the MS2 level with *Q*-value data filtering and cross-run normalization with automatic row selection.

Volcano plots were made using ggplot2 (*62*) in R (*63*), and mitochondrial genes were highlighted on the basis of mitochondrial processes derived from OmicsVolcano. Proteins forming components of the OXPHOS complexes were annotated and visualized by Pathvisio (*64*) with WikiPathways (*65*). Gene ontology analyses were performed on significantly changing proteins (adjusted *P* value < 0.05 and absolute log_2_ fold change > 0.5) using PANTHER 17.0 (*66*) for biological process and cellular compartment pathways.

### Gel electrophoresis and immunoblotting

Fifty micrograms of cell lysates from CAL51 cells was separated on 4 to 12% Bis-Tris gels (Invitrogen), and samples were transferred onto a polyvinylidene difluoride (PVDF) membrane (Bio-Rad). Specific proteins were detected using mouse antibodies against β-actin (ab6276, Abcam, diluted 1:1000) and rabbit antibody against STARD7 (15689-1-AP, Proteintech, diluted 1:1000). IRDye 680RD goat anti-mouse IgG (926-68070) and IRDye 800CW (926-32211) goat anti-rabbit IgG secondary antibodies (Li-COR Biosciences, diluted 1:10,000) were used to detect primary antibodies. Blots were visualized using an infrared imaging system (Li-COR biosciences).

### Antibodies

Details of all antibodies used in this study, including their sources, catalog numbers, and dilutions, are provided in table S5.

### Intracellular amino acid analysis

Amino acids were extracted in 50:50 water/methanol by cell lysis (of 2 million cells) or mitochondrial lysis (5 mg) from the control and knockout cell lines. The solvent was analyzed by targeted reversed-phase high-speed quantitative ultraperformance liquid chromatography–tandem mass spectrometry (UPLC-MS) analysis as previously described (*17*).

### Cell culture amino acid uptake and efflux rate analysis

Approximately 63,000 cells were seeded and grown as above in triplicate and sampled at 24, 48, 72, and 96 hours. Samples were centrifuged, and the supernatant was diluted with Milli-Q water (1:2) and then further mixed (1:1) with an internal standard of sarcosine and 2-aminobutanoic acid. Amino acids were derivatized and analyzed with an Agilent 1200-SL HPLC system with a fluorescence detector (FLD) (catalog no. G1321A; Agilent) as described previously (*67*). Uptake and efflux rates were estimated by calculating the slope of the pooled concentration of amino acids over time measurements for all replicates. Statistical significance was determined by performing a *t* test to compare the slopes of the regressions, and the Benjamini-Hochberg procedure was used to correct for multiple testing, with an adjusted *P* value of < 0.05 assumed as significant.

### Expression of SLC25A48 in *S. cerevisiae*

The gene encoding SLC25A48 (UniProt accession code: Q6ZT89) was codon optimized and synthesized (GenScript). To aid expression and purification, the gene was synthesized with an N-terminal extension (GKPRTSPK corresponding to residues 14 to 21 of the human oxoglutarate carrier; UniProt accession code: Q02978) and an eight-histidine tag and factor Xa protease cleavage site. These modifications do not affect any important structural or functional elements of the carrier (*7*, *32*). This construct was cloned into the pYES2/CT vector (Invitrogen) and transformed into the protease-deficient yeast strain BJ2168 (*68*). Successful transformants were selected on SC-Ura + 2% (w/v) glucose plates. For large-scale expression, a preculture of cells grown in SC- Ura + 2% (w/v) glucose was inoculated into 50 liters of YPG + 0.1% glucose medium in an Applikon Pilot Plant 140-liter bioreactor. Cells were grown at 30°C for 20 hours, induced with 2% galactose, grown for 8 hours, and harvested by centrifugation (4000*g*, 20 min, 4°C). Crude mitochondria were prepared using a bead mill (Dyno-Mill Multilab, Willy A. Bachofen AG) by established methods (*68*).

### Purification of SLC25A48 by nickel affinity chromatography

Crude mitochondria were solubilized in a solution containing 1.5% (w/v) lauryl maltose neopentyl glycol, an EDTA-free protease inhibitor tablet (Roche), 40 mM imidazole, and 150 mM NaCl. The mixture was mixed by rotation at 4°C for 1 hour, and the soluble fraction separated from insoluble material by centrifugation (200,000*g*, 45 min, 4°C). Nickel Sepharose (GE Healthcare), at a volume of 0.7 ml of slurry per 1 g of crude mitochondria, was added to the supernatant. This mixture was stirred at 4°C for 1.5 hours and dispensed into an empty PD-10 column, and the settled resin washed with 40 column volumes of buffer A [20 mM Hepes (pH 7.0), 150 mM NaCl, 80 mM imidazole, tetraoleoyl cardiolipin (0.1 mg/ml), and 0.1% (w/v) lauryl maltose neopentyl glycol], followed by 10 column volumes of buffer B [20 mM Hepes (pH 7.0), 150 mM NaCl, 10 mM imidazole, 5 mM CaCl_2_, tetraoleoyl cardiolipin (0.1 mg/ml), and 0.1% (w/v) lauryl maltose neopentyl glycol]. The resin was resuspended with 0.7 ml of buffer B into a final volume of 1.5 ml and incubated with 7.5 μg of factor Xa protease (NEB) and on-column cleavage performed overnight at 4°C with rotation. The protein was collected by centrifugation (5000*g*, 2 min, 4°C) and passed through a vivapure IEX Q Mini H column, pre-equilibrated with buffer B (3000*g*, 2 min, 4°C). The sample was desalted using a midi PD10 desalting column (GE Healthcare), pre-equilibrated with buffer C [20 mM Hepes (pH 7.0), 100 mM NaCl, tetraoleoyl cardiolipin (0.1 mg/ml), and 0.1% (w/v) lauryl maltose neopentyl glycol]. The protein concentration was measured by spectrometry (NanoDrop Technologies) at 280 nm (extinction coefficient: 40,260 M^−1^ cm^−1^; protein mass: 34,340 Da). The purity and stability of the final samples were assessed by gel electrophoresis and thermostability shift assays.

### Thermostability analysis

Differential scanning fluorimetry (nanoDSF) was used to assess the thermostability of protein. In this study, the protein was characterized by its tryptophan and tyrosine content (3 tryptophans and 13 tyrosines). For the analysis, 1 μg of protein was mixed with or without 10 mM substrate. NanoDSF-grade standard glass capillaries were used to load samples into the Prometheus NT.48. The temperature was progressively increased from 25° to 95°C, at a rate of 4°C/min. The software PR.ThermControl (NanoTemper Technologies) was used to determine the apparent melting temperatures (*T*_m_). The change in melting temperature (∆*T*_m_) upon addition of compound was calculated by subtracting the apparent *T*_m_ in the absence of compound from the apparent *T*_m_ in the presence of compound. A positive ∆*T*_m_ indicates that a compound binds to and stabilizes the protein.

### Preparation of lipids for protein purification

Tetraoleoyl cardiolipin (18:1) (Avanti Polar Lipids) was dissolved in a 10% (w/v) solution of lauryl maltose neopentyl glycol (Anatrace) by vortexing for 2 hours, resulting in a lipid concentration of 10 mg/ml in a 10% (w/v) detergent solution, and stored in liquid nitrogen.

### Lipidomic analysis

Lipid extraction and derivation was performed as described previously (*18*). For each CL LC-MS/MS experiment, 2 μl of the above extract was loaded onto an ACQUITY UPLC HSS T3 column [100 Å, 1.8 μm, 1 mm by 150 mm (Waters)] at a rate of 0.2 μl/s and a dispense speed of 2 μl/s. The analytical column was maintained at 50°C. A 33-min LC gradient separation was performed using a Vanquish NEO UHPLC system (Thermo Fisher Scientific) at a flow rate of 0.06 ml/min. The mobile phases comprised solvent A (40% acetonitrile, 60% H_2_O, 10 mM ammonium acetate, and 5 μM medronic acid) and solvent B (90% isopropanol, 10% acetonitrile, and 10 mM ammonium acetate). The gradient started at 3% B for 5 min, followed by an increase to 70% B over 5 min, then to 99% B over 16 min, maintained at 99% B for 3 min, and finally returned to 3% B for a 4-min stabilization before subsequent runs.

Mass spectra were acquired using a Thermo Fisher Scientific Orbitrap Fusion Lumos Tribrid mass spectrometer. The ion source parameters were applied as follows: The negative ion voltage was set to 3 kV; the sheath gas and aux gas and sweep gas rate was set to 25, 5, and 0, respectively; the ion transfer tube was set at 300°C; and the vaporizer temperature set at 150°C. Spectra were recorded in negative ionization mode. For MS1 acquisitions, data were collected at a mass resolving power of 120,000, with a scan range of 350 to 2000 *m/z* from 0 to 21.5 min and 1200 to 2000 *m/z* from 21.5 to 33 min. MS/MS data were acquired at a mass resolving power of 15,000 with HCD energy set to 25 and a quadrupole isolation window of 1 *m/z*. From 0 to 21.5 min, MS ions with intensities above 2 × 10^4^ were selected as precursors for MS/MS analysis. Dynamic exclusion was applied after one occurrence, with an exclusion duration of 15 s. The MS/MS maximum injection time was set to auto mode, and the automatic gain control (AGC) target was set to standard. From 21.5 to 33 min, MS ions with intensities above 4 × 10^4^ were selected as precursors for MS/MS analysis. Dynamic exclusion was applied after three occurrences, with an exclusion duration of 15 s. During this period, the MS/MS maximum injection time was set to 22 ms, and the AGC target was set to 100%. Raw spectra were converted to mzml format using MS Convert (*69*). CL peaks was analyzed using MS Dial 5.2.240424.3 (*70*) with the following settings: MS1 tolerance of 0.01 Da, MS2 tolerance of 0.025 Da, profile peak detection, alignment tolerances of 0.1 min and 0.015 Da, and using the native MSDial identification database. Semiquantitative analysis was performed by normalizing species to an internal standard of 14:0 CL (CL 56:0, Avanti Polar Lipids).

### Statistical analyses

Values are means ± SD of biological replicates. A two-way Student’s *t* test was used for most analyses assuming normal distribution unless otherwise stated, there was no blinding of data, biological replicates were used for all experiments, and the sample size is included in the figure legends. Randomization was not carried out and samples were not excluded from the analyses.
