# Supplementary material for: Specific SLC25 carriers regulate mitochondrial protein synthesis
Source: Sci Adv. 2026 Feb 25;12(9):eaeb0049. doi: 10.1126/sciadv.aeb0049 (PMC12935053; doi:10.1126/sciadv.aeb0049)
Supplement: Supplementary file 1 — Figs. S1 to S7 Legends for tables S1 to S4 Table S5 [file sciadv.aeb0049_sm.pdf]

Science Advances
AAAS

Supplementary Materials for

Specific SLC25 carriers regulate mitochondrial protein synthesis

Danielle L. Rudler *et al.*

Corresponding author: Oliver Rackham, oliver.rackham@curtin.edu.au;
Aleksandra Filipovska, aleksandra.filipovska@uwa.edu.au

Sci. Adv. **12**, eaeb0049 (2026)
DOI: 10.1126/sciadv.aeb0049

The PDF file includes:

Figs. S1 to S7
Legends for tables S1 to S4
Table S5

Other Supplementary Material for this manuscript includes the following:

Tables S1 to S4

Supplementary Information

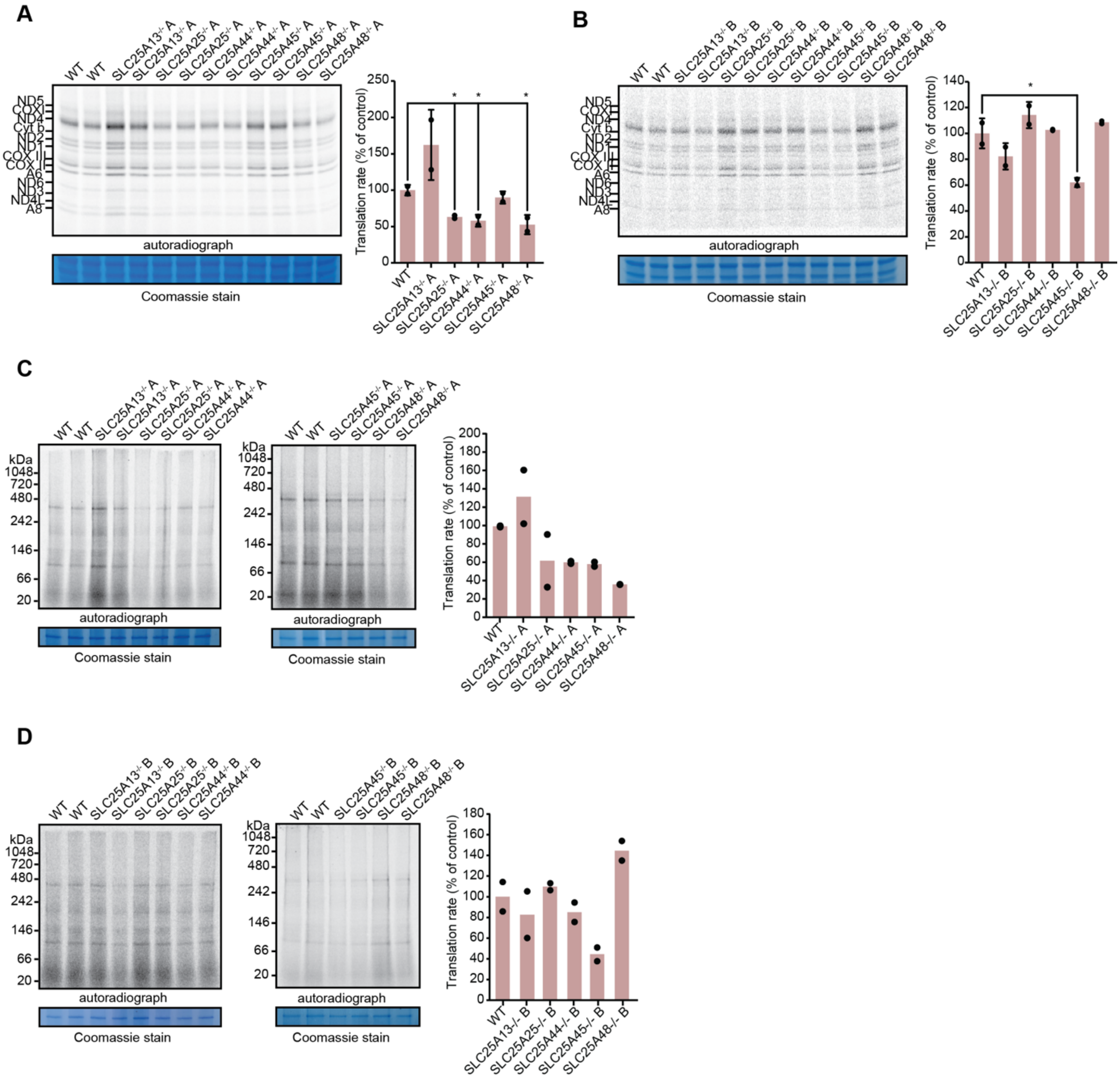

Fig. S1, related to Fig. 1. Transient knockouts of SLC25 proteins affect mitochondrial translation rates.

Mitochondrial *de novo* protein synthesis measured in transient knockout cell lines of SLC25 members with two guides: gRNA-A **(A)** and gRNA-B **(B)** compared to controls (WT), and BN-PAGE translation of the same SLC25 transient knockout cells with gRNA-A **(C)** and gRNA-B **(D)**. Cells were labelled by pulse incorporation of ^{35}S-labelled methionine and cysteine. Equal amounts of protein were separated by SDS-PAGE, visualised by

autoradiography, and quantitated using Image J. Data are mean values ± SD of n = 3 biological replicates. *p<0.05, **p<0.01, two-tailed Student's *t*-test assuming unequal variances.

Supplementary Information

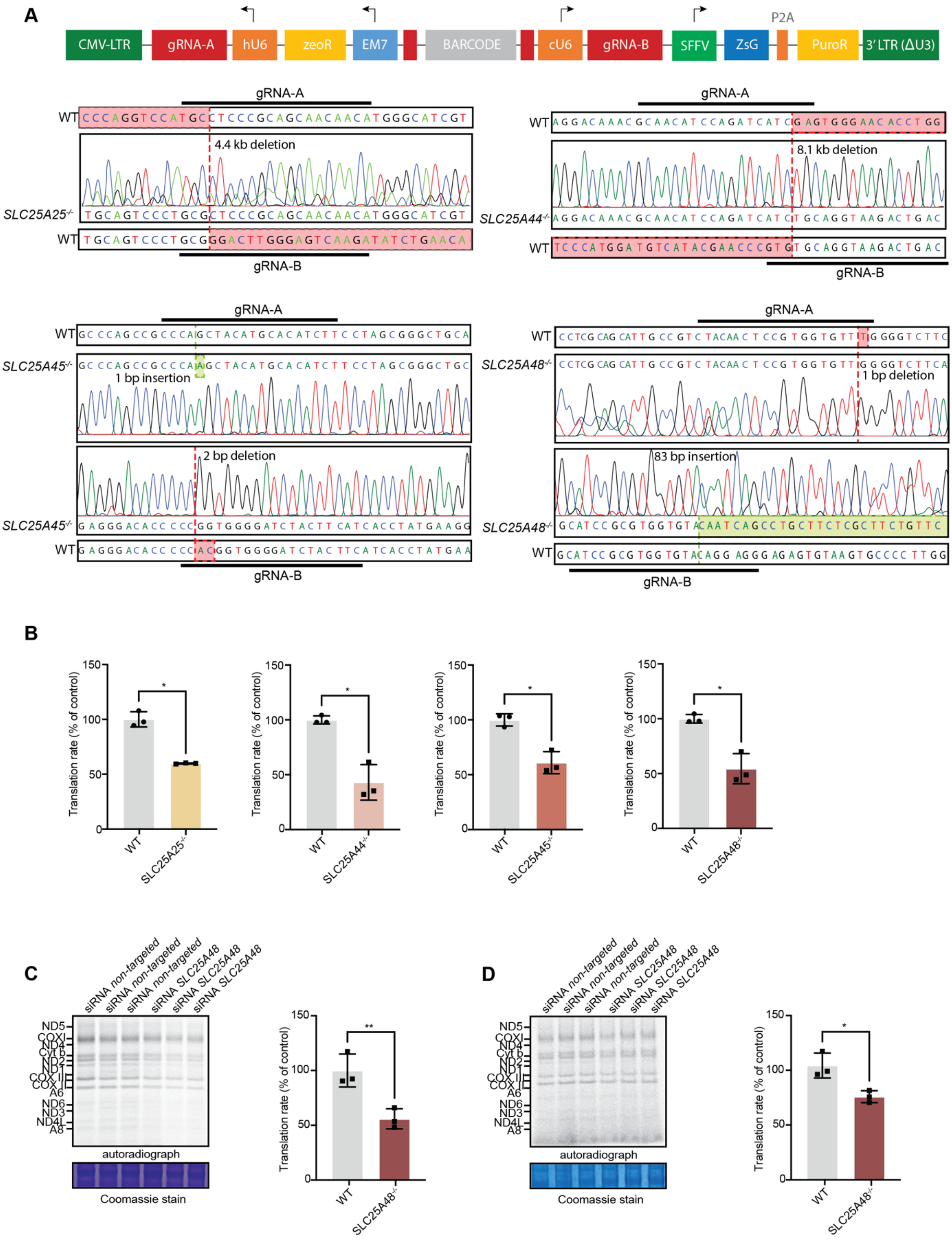

Fig. S2, related to Fig. 1. Generation of SLC25 knockout cell lines.

Supplementary Information

(A) Schematic representing the structure of the plasmids used to generate SLC25 knockouts. Single cell sorting was performed following transfection with plasmids containing gRNA expression cassettes. Sequencing chromatograms illustrate the knockout sequences for each transporter with gRNA-A and gRNA-B edit positions annotated. **(B)** Quantitation of the translation autoradiographs in Figure 1D. **(C)** CAL51 or HeLa **(D)** cells were treated with non-targeting or *SLC25A48* directed siRNAs for 72 h and labelled by pulse incorporation of ^{35}S-labelled methionine and cysteine. Equal amounts of protein were separated by SDS-PAGE, visualised by autoradiography and quantitated using Image J. Data are mean values ± SD of n = 3 biological replicates. *p<0.05, **p<0.01, two-tailed Student's *t*-test assuming unequal variances.

Supplementary Information

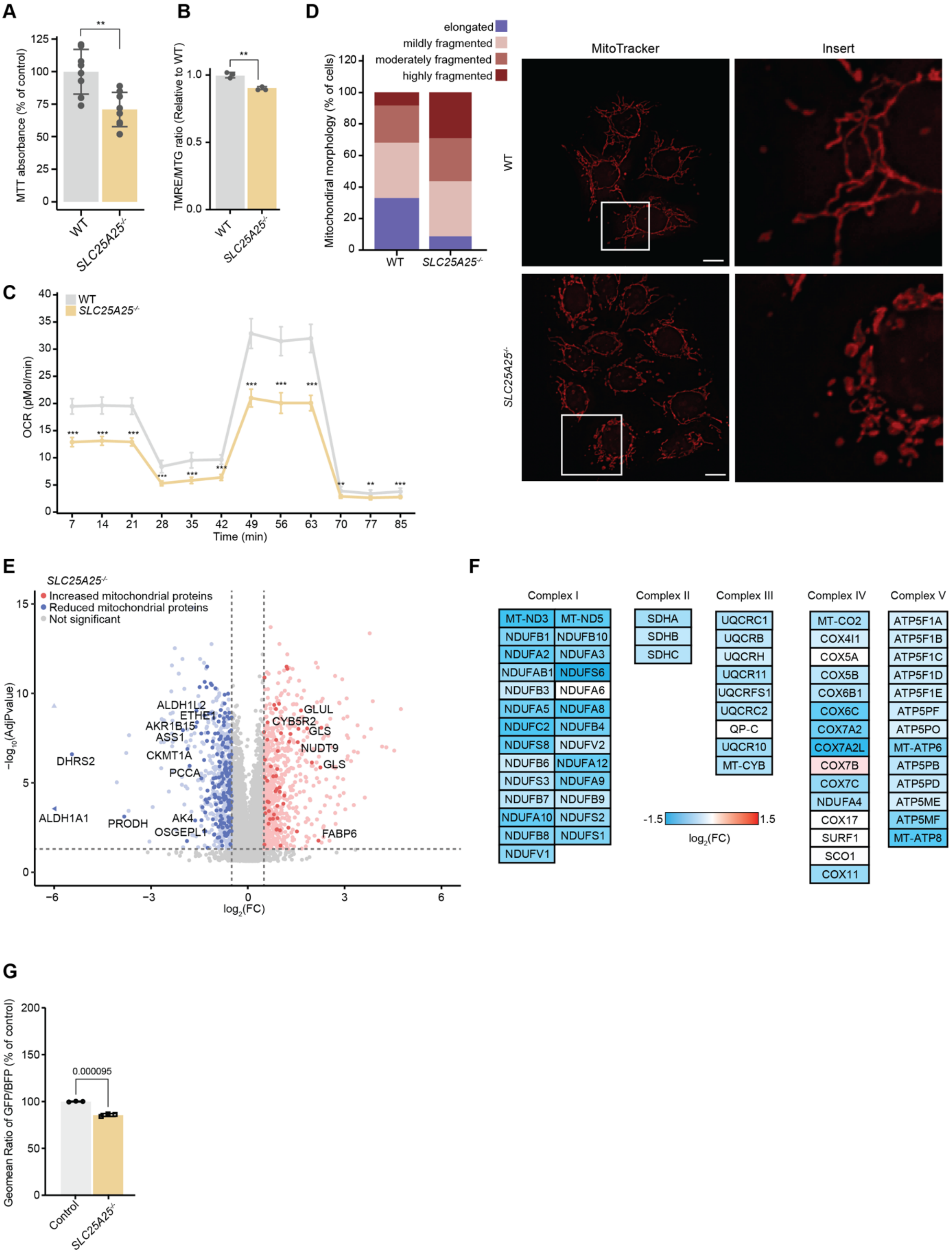

Fig. S3, related to Figs. 1 and 2. Loss of SLC25A25 leads to changes in protein expression, OXPHOS complexes and mitochondrial function.

Supplementary Information

(A) Changes in cellular proliferation measured via MTT absorbance in control (WT) and *SLC25A25*$^{-/-}$ knockout cells. **(B)** Tetramethylrhodamine (TMRE) was used to measure $\Delta\Psi_m$ and data were normalized to mitochondrial content using the $\Delta\Psi_m$ -insensitive dye MitoTracker Green (MTG), graphed as the TMRE/MTG ratio. Data are mean values ± SD of n = 3 biological replicates. **p<0.01, paired two-way student *t* test. **(C)** Oxygen consumption rate measured using the Seahorse XF Cell Mito Stress Test kit for control (WT) and *SLC25A25*$^{-/-}$ cells. **(D)** Mitochondrial morphology of SLC25A25 knockout cells compared to controls with MitoTracker Orange and visualised by fluorescence microscopy. Inserts are representative of white square encompassed areas. Scale bar represents 10 μm. Qualitative scoring of the morphology in n>50 images for control and knockout cells. Details of morphological classifications are described in the Methods. **(E)** Proteomic analysis of *SLC25A25*$^{-/-}$ cells compared to controls shown as a volcano plot. Significantly changing proteins (adjusted p-value < 0.05) with absolute $\log_2$ fold change > 0.5 are indicated in light blue and red, and significantly changing mitochondrial proteins are indicated in dark blue and red. Mitochondrial proteins involved in metabolism, oxidative phosphorylation or translation with absolute $\log_2$ fold change > 1.5 are labelled by text. **(F)** Significant proteomic results (adjusted p-value < 0.05) of the subunits of the mitochondrial OXPHOS complexes coloured by $\log_2$ fold change. **(G)** Quantitation of mitochondria-ER interactions in control and *SLC25A25*$^{-/-}$ cells using a TOMM20-VAPB reporter. Data are presented as mean ± SD of two biologically independent experiments (each performed with 3 replicates per genotype per experiment). Student's paired *t* test p values are indicated on the graphs.

Supplementary Information

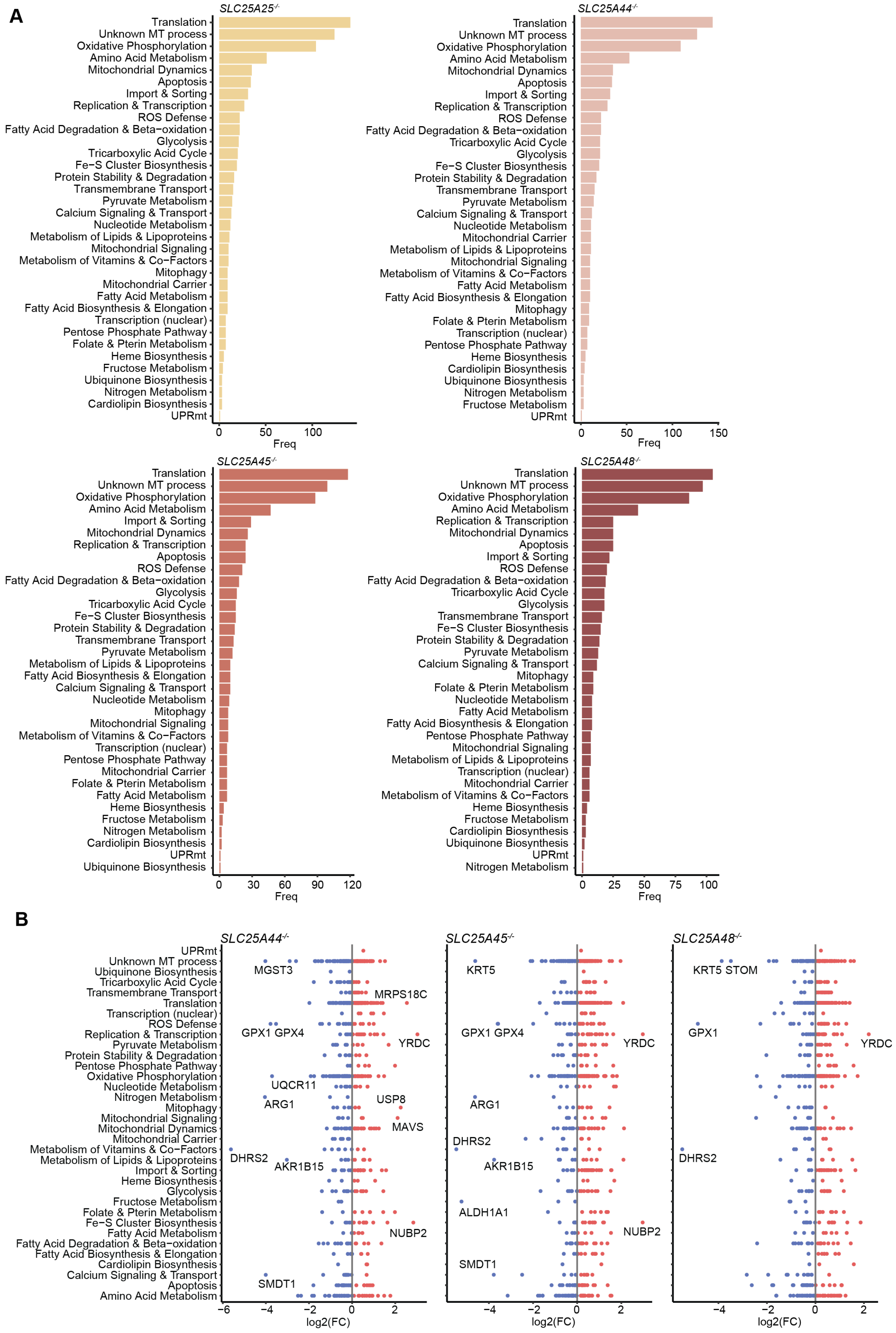

Supplementary Information

Fig. S4, related to Fig. 2. Mitochondrial proteomic changes in knockout SLC25 cells.

(A) Bar graphs of the categories of mitochondrial proteins obtained from Mitocarta and OmicsVolcano with the number of proteins that are significantly changing (adjusted p-value < 0.05). **(B)** Distribution of $\log_2$ fold change of significantly changing mitochondrial proteins (adjusted p-value < 0.05) in each category obtained from Mitocarta and OmicsVolcano. Red indicates increased steady state levels and blue coloured points indicate a decrease.

Supplementary Information

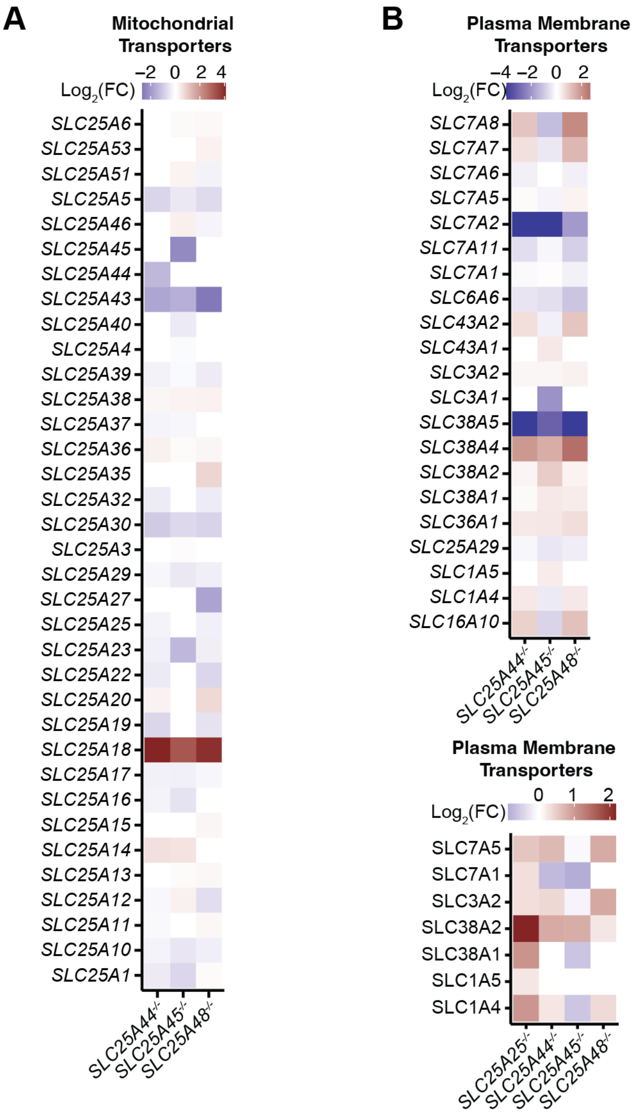

Fig. S5, related to Fig. 5. Loss of SLC25A44, SLC25A45 and SLC25A48 leads to changes in mitochondrial and plasma transporters.

Significantly changing (adjusted p-value < 0.05 and absolute $\log_2$ fold change > 0.5) transcripts encoding mitochondrial **(A)** and plasma **(B)** transporters detected by RNA-seq in *SLC25A44*$^{-/-}$, *SLC25A45*$^{-/-}$ and *SLC25A48*$^{-/-}$ cells.

Supplementary Information

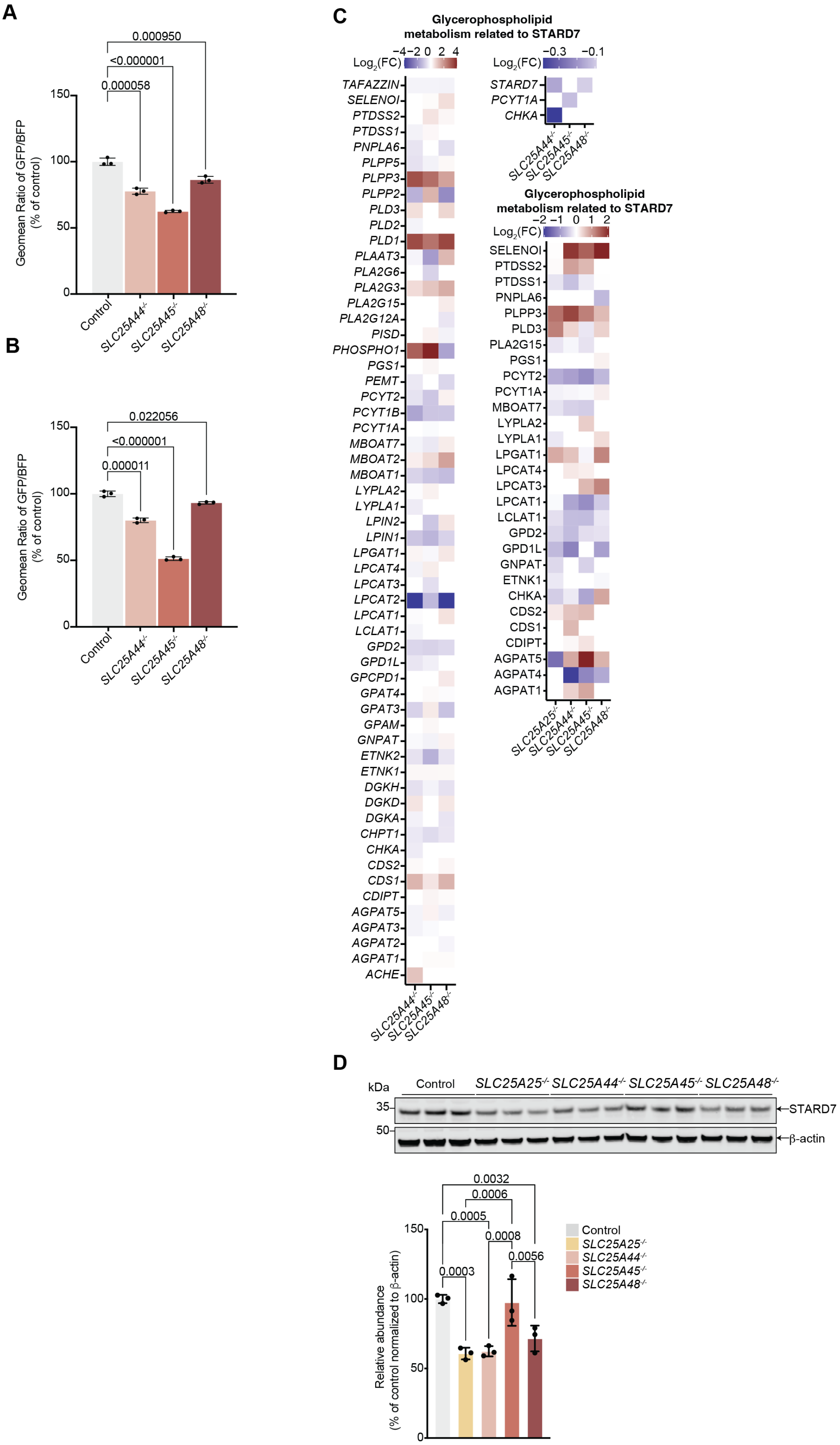

Supplementary Information

Fig. S6 related to Fig. 6. Loss of mitochondrial transporters reduces mitochondria-ER contact sites and perturbs glycerophospholipid metabolism.

Quantitation of mitochondria-ER interactions in control and knockout cell lines using SPLICS reporters with short **(A)** and long **(B)** linkers. Data are presented as mean ± SD of two biologically independent experiments (each performed with 3 replicates per genotype per experiment). Student's paired *t* test p values are indicated on the graphs. **(C)** Significantly changing (adjusted p-value < 0.05 and absolute $\log_2$ fold change > 0.5) transcripts encoding glycerophospholipid metabolism enzymes and proteins detected by RNA-seq or mass spectrometry, respectively, in *SLC25A44*$^{-/-}$, *SLC25A45*$^{-/-}$ and *SLC25A48*$^{-/-}$ cells. **(D)** 50 µg of cell lysates were resolved by SDS-PAGE and immunoblotted for STARD7. GAPDH was used as a loading control. Data are mean values ± SD of n = 3 biological replicates, paired two-way student *t* test, p values are shown on the graph.

Supplementary Information

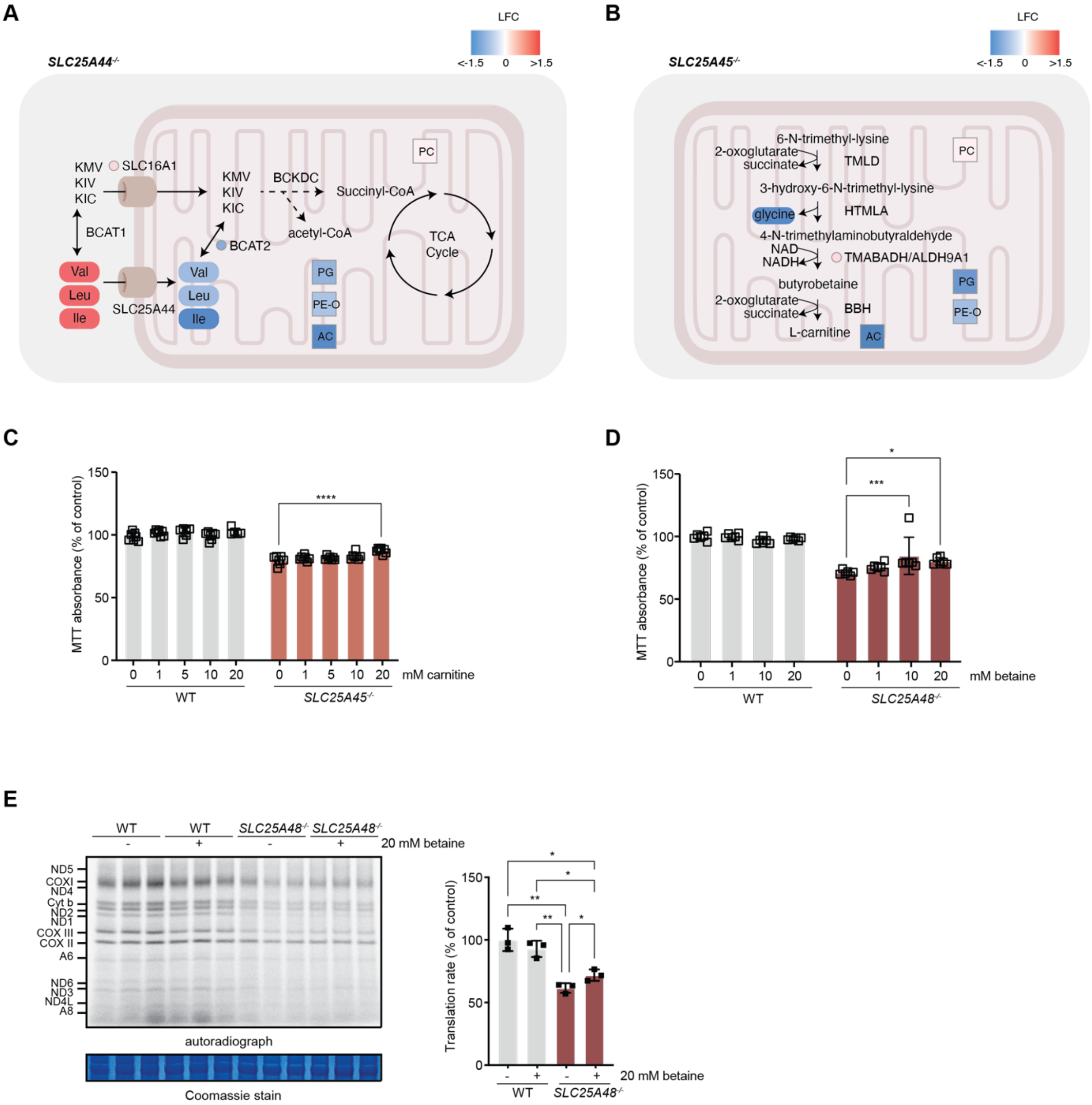

Fig. S7, related to Fig. 6. The mitochondrial carriers SLC25A44 and SLC25A45 regulate mitochondrial protein synthesis via import of BCAA or TMA and DML. Significantly changing proteomic, metabolomic and lipidomic (adjusted p-value < 0.05) changes mapped onto mitochondrial pathways in the *SLC25A44*$^{-/-}$ cells **(A)** and the *SLC25A45*$^{-/-}$ cells **(B)**. Changes in cellular proliferation measured using the MTT assay in control (WT), *SLC25A45*$^{-/-}$ **(C)** and *SLC25A48*$^{-/-}$ **(D)** cells treated with increasing concentrations of carnitine or betaine, respectively. Data are mean values ± SD of n = 3 biological replicates. *p<0.01, ***p<0.001, ****p<0.0001, a one-way ANOVA with Tukey post hoc analysis for multiple

comparisons. **(E)** Mitochondrial *de novo* protein synthesis measured by pulse incorporation of ^{35}S-labelled methionine and cysteine in control (WT) and *SLC25A48*$^{-/-}$ knockout cells grown in the presence or absence of 20 mM betaine for 72 h. Equal amounts of protein were separated by SDS-PAGE, visualised by autoradiography and quantitated using Image J. Data are mean values ± SD of n = 3 biological replicates. *p<0.05, **p<0.01, a one-way ANOVA with Tukey post hoc analysis for multiple comparisons.

Supplementary Information

Table S1. RNAseq differential expression and gene ontologies for *SLC25A44*$^{-/-}$, *SLC25A45*$^{-/-}$ and *SLC25A48*$^{-/-}$ cells compared to control cells.

Table S2. Proteomic differential changes and gene ontologies for *SLC25A25*$^{-/-}$, *SLC25A44*$^{-/-}$, *SLC25A45*$^{-/-}$ and *SLC25A48*$^{-/-}$ cells compared to control cells.

Table S3. Metabolite levels in cells and isolated mitochondria from control (WT), *SLC25A25*$^{-/-}$, *SLC25A44*$^{-/-}$, *SLC25A45*$^{-/-}$ and *SLC25A48*$^{-/-}$ cells.

Table S4. Lipid levels in cells and isolated mitochondria from control (WT), *SLC25A25*$^{-/-}$, *SLC25A44*$^{-/-}$, *SLC25A45*$^{-/-}$ and *SLC25A48*$^{-/-}$ cells.

Table S5. Details of antibodies used in this study, including sources, catalog numbers, and dilutions.

**Antibody**	**Source, Purity and validation**	**Catalog number**	**Dilution**
Mouse anti β-actin	Abcam, affinity purified, WB validated in β-actin knockout HAP1 cells	ab6276	1:1,000 WB
Rabbit anti-STARD7	Proteintech, antigen affinity purification, WB validated	15689-1-AP	1:1,000 WB
Goat anti-rabbit IRDye 800CW	LICOR, affinity purification, WB validated	926-32211	1:10,000 WB

Supplementary Information

Goat anti-mouse IRDye 680RD	LICOR, affinity purification, WB validated	926-68070	1:10,000 WB
**Cells**	**Validation**	**Purity**	**Source**
CAL51-mCherry-Cas9	STR Validated	Mycoplasma free	Gift from Dr Vihandha Wickramasinghe
SLC25A44$^{-/-}$ CAL51 cells	STR Validated	Mycoplasma free	Produced in this study
SLC25A45$^{-/-}$ CAL51 cells	STR Validated	Mycoplasma free	Produced in this study
SLC25A48$^{-/-}$ CAL51 cells	STR Validated	Mycoplasma free	Produced in this study
